# Reactive Oxygen Species‐Regulating Strategies Based on Nanomaterials for Disease Treatment

**DOI:** 10.1002/advs.202002797

**Published:** 2020-12-20

**Authors:** Chenyang Zhang, Xin Wang, Jiangfeng Du, Zhanjun Gu, Yuliang Zhao

**Affiliations:** ^1^ CAS Key Laboratory for Biomedical Effects of Nanomaterials and Nanosafety Institute of High Energy Physics Chinese Academy of Sciences Beijing 100049 China; ^2^ College of Materials Science and Optoelectronic Technology University of Chinese Academy of Sciences Beijing 100049 China; ^3^ Department of Medical Imaging Shanxi Medical University Taiyuan 030001 China; ^4^ CAS Center for Excellence in Nanoscience National Center for Nanoscience and Technology of China Chinese Academy of Sciences Beijing 100190 China; ^5^ GBA Research Innovation Institute for Nanotechnology Guangdong 510700 China

**Keywords:** nanomaterials, reactive oxygen species, ROS generation, ROS scavenger, therapy

## Abstract

Reactive oxygen species (ROS) play an essential role in physiological and pathological processes. Studies on the regulation of ROS for disease treatments have caused wide concern, mainly involving the topics in ROS‐regulating therapy such as antioxidant therapy triggered by ROS scavengers and ROS‐induced toxic therapy mediated by ROS‐elevation agents. Benefiting from the remarkable advances of nanotechnology, a large number of nanomaterials with the ROS‐regulating ability are developed to seek new and effective ROS‐related nanotherapeutic modalities or nanomedicines. Although considerable achievements have been made in ROS‐based nanomedicines for disease treatments, some fundamental but key questions such as the rational design principle for ROS‐related nanomaterials are held in low regard. Here, the design principle can serve as the initial framework for scientists and technicians to design and optimize the ROS‐regulating nanomedicines, thereby minimizing the gap of nanomedicines for biomedical application during the design stage. Herein, an overview of the current progress of ROS‐associated nanomedicines in disease treatments is summarized. And then, by particularly addressing these known strategies in ROS‐associated therapy, several fundamental and key principles for the design of ROS‐associated nanomedicines are presented. Finally, future perspectives are also discussed in depth for the development of ROS‐associated nanomedicines.

## Graphical abstract

Reactive Oxygen Species (ROS)‐regulating nanomedicines for disease treatments mainly include ROS‐upregulating nanomedicines and ROS‐downregulating nanomedicines. Here, ROS‐upregulating nanomedicines can exert the toxic effect by employing nanoplatforms to enhance ROS generation in pathological sites for ROS‐induced toxic therapy, and ROS‐downregulating nanomedicines can scavenge excess ROS to maintain normal physiological process and avoid oxidative stress injuries for antioxidant therapy.

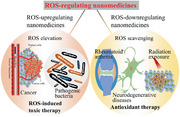

## 1 Introduction

### 1.1 ROS in Living Organisms

Reactive oxygen species (ROS) is a general term used to describe the species of highly active radicals formed upon unpaired electrons of oxygen such as hydroxyl radical (^•^OH) and superoxide (^•^O_2_^−^). The term ROS is most often expanded to include reactive oxygen‐containing compounds or nonradical oxidizing agents such as singlet oxygen (^1^O_2_), ozone (O_3_), hydrogen peroxide (H_2_O_2_), and hypochlorous acid (HOCl).^[^ ^1^, ^2^ ^]^ The presence of free radicals has been found in chemistry since the early 20th century and in biological systems since 1954.^[^ ^3^, ^4^ ^]^ ROS can come from different pathways such as the photolysis of gaseous ozone, materials‐mediated catalytic reactions, endogenous activities in biological systems.^[^ ^5^, ^6^, ^7^, ^8^ ^]^ In recent years, ROS in living organisms become one of the most important research fields because ROS play a vital role in adjusting various physiological functions. In general, sources of ROS in living organisms can be divided into exogenous and endogenous sources. The exogenous sources of ROS are represented by exposure to engineered nanoparticles (NPs), radiation, chemotherapeutics, and microbial infection.^[^ ^9^, ^10^, ^11^, ^12^ ^]^ Endogenous ROS can be produced from the cellular respiration and normal metabolism.^[^ ^13^ ^]^ And all high‐concentration ROS are extremely toxic to living organisms. Nevertheless, in normal physiological processes, ROS are well‐known and well‐described messengers in various cellular functions, which can be identified as a signal molecule or a regulator in living systems.^[^ ^14^ ^]^ Here, it is believed that the effect of ROS on physiological processes is attributed to their capabilities to alter the activity of specific proteins.^[^ ^15^ ^]^ In the last few decades, the roles of ROS in normal physiological processes have been widely studied including blood vessel modulation, immune function, oxygen sensing, gene activation, and cellular growth.^[^ ^16^, ^17^ ^]^ Besides the roles in normal cell physiological function, ROS have also been implicated in the initiation and development of pathological processes involving aging, cancer, insulin resistance, diabetes mellitus, cardiovascular diseases, and Alzheimer's disease, etc.^[^ ^18^ ^]^ Over‐expressed ROS can be observed in those diseases, which may induce tissue dysfunction or cell death. As a result, a stable concentration of ROS can serve as a messenger in regulating physiological processes, while excessive ROS generation can exert their toxicity to trigger tissue dysfunction or cell death. Therefore, guaranteeing the redox homeostasis in living organisms has great significance to keep the normal physiological functions and reduce the incidence of diseases. In general, cellular redox homeostasis is maintained by antioxidant‐protective systems such as superoxide dismutase (SOD), catalase (CAT), peroxidase (POD), glutathione (GSH), vitamin C, vitamin E, and so forth.^[^ ^19^ ^]^

### 1.2 ROS‐Related Nanomedicines for Therapy

**Figure 1:**
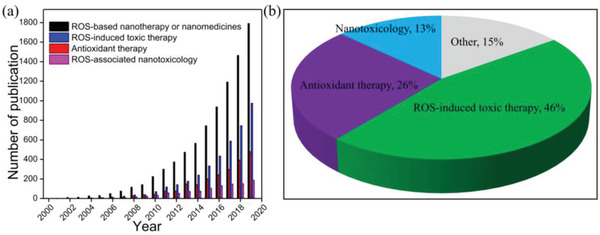
a) Number of ROS‐based nanotherapeutic modalities or nanomedicine publications worldwide according to the Web of Science Core Collection. b) Pie chart of ROS‐based nanotherapeutic modalities or nanomedicine publications in the field of ROS‐associated antioxidant therapy, ROS‐induced toxic therapy, and ROS‐associated nanotoxicology.

Whether ROS act as damage molecules or signal molecules depends on the concentration of ROS in living organisms, which is associated with the endogenous antioxidant‐protective systems. Therefore, research on the regulation of ROS concentration has caused wide concern, mainly involving the topics in ROS‐regulating therapy such as antioxidant therapy triggered by ROS scavengers and ROS‐induced toxic therapy mediated by ROS‐elevation agents (e.g., photosensitizers (PSs)). Currently, ROS‐regulating researches have achieved great development, offering reasonable explanations on the physiological and pathological roles of ROS.^[^ ^5^, ^20^, ^21^, ^22^ ^]^ In particular, over the past few decades, nanoscience and nanotechnology are introduced in ROS‐regulating study, further accelerating their fast development. ROS research with the assistance of nanotechnology is mainly reflected in the development of ROS‐related nanomedicines or nanotherapeutic modalities. Here, these ROS‐based nanotherapeutic modalities or nanomedicines that can regulate ROS progress depend on the intrinsic biophysical and biochemical characteristics of nanomaterials, such as their appropriate sizes (usually 10–100 nm), many interface/surface options and high specific surface area.^[^ ^23^ ^]^ The employment of nanomaterials in ROS‐regulating therapy exhibits various advantages such as improved stability and biocompatibility of ROS‐regulating agents, enhanced drug accumulation, optimized pharmacokinetics, and so on. Inspired by the great achievements on ROS‐related nanotherapeutic modalities or nanomedicines, an overview in the field is important and necessary, which may provide new possibilities for the further development of ROS‐based nanoresearch. In order to have an objective analysis and a deep insight into the research status and current concerns about nanomaterials‐mediated ROS research in biological system, first, we used keyword searches for the defined ROS‐based nanotherapeutic modalities or nanomedicines in the field of ROS‐associated antioxidant therapy, ROS‐induced toxic therapy and ROS‐associated nanotoxicology to search the publications in the Web of Science Core Collection database, trying to figure out the hotspot and keystone of the studies on nanomaterials‐mediated ROS study (Supporting information). According to the data from the Web of Science Core Collection, the bibliometrics documents showed that nanomedicine research has exploded and gained strong momentum worldwide in the past few decades, and the number of publications in ROS‐related nanodrug or therapy is rapidly growing, rising from several articles per year in the late 1990s to more than 1700 in the year of 2019 (**Figure** **1**a). In this field, the ROS‐induced toxic therapy (e.g., photodynamic therapy (PDT), sonodynamic therapy (SDT), radiotherapy (RT), chemodynamic therapy (CDT)) is the dominant research topic, accounting for 46% of the publications. And the ROS‐associated antioxidant therapy is another important part of the field with a publication share of 26% (Figure 1b). The result indicates that antioxidant therapy and toxic therapy are at the forefront of nanomaterials‐mediated ROS research.

### 1.3 Concern and Study in This Review

**Figure 2:**
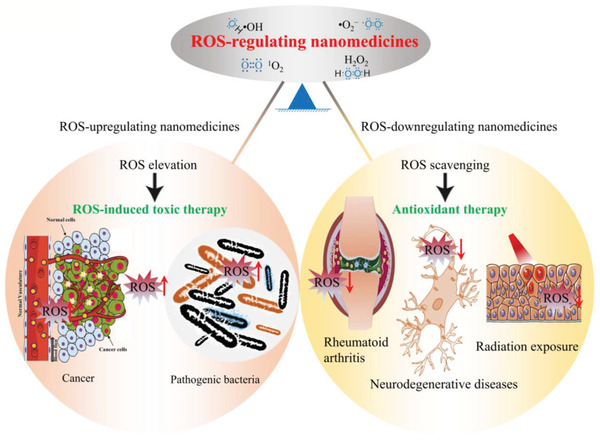
ROS‐regulating nanomedicine for ROS‐induced toxic therapy and antioxidant therapy. The ROS‐upregulating nanomedicine with unique ROS‐elevation ability for the treatment of various pathological disfunctions such as cancer and bacterial infection, etc. Adapted with permission.^[^ ^300^ ^]^ Copyright 2018, Springer Nature. The ROS‐downregulating nanomedicine with unique ROS‐scavenging ability for the treatment of various pathological disfunctions such as rheumatoid arthritis, neurodegenerative diseases and radiation exposure‐induced injury, etc. Adapted with permission.^[^ ^301^ ^]^ Copyright 2017, Elsevier B.V. Adapted with permission.^[^ ^302^ ^]^ Copyright 2018, American Chemical Society.

The bibliometric statistics of ROS‐associated nanomedicine research reveals that antioxidant therapy and ROS‐induced toxic therapy indeed occupy the research center of ROS‐related nanomedicine. Despite great achievements have been made in ROS‐related nanomedicines for disease treatments, we notice that the research in the past decades undertaken by the biomaterials research community is primarily focused on some common issues such as the synthesis of nanomaterials with novel structure and function, the characterization of nanomaterials, and the discovery of interesting active pathway. While some fundamental but key questions such as the rational design principle for ROS‐related nanomaterials are held in low regard. For the design principle of ROS‐related nanomedicines, it can provide the initial framework for technicians and scientists to design and optimize the ROS‐regulating nanomedicines, thereby minimizing the gap of nanomedicines for biomedical application during the design stage. Therefore, in order to promote the stable development and deep research of ROS‐related nanomedicines, an overview of the current progress of ROS‐associated nanomedicines in disease therapy involving antioxidant therapy and ROS‐induced toxic therapy will be summarized (**Figure** **2**). Meanwhile, the key therapeutic mechanisms of ROS‐based nanomedicine are highlighted. And then, by particularly addressing these known strategies in ROS‐associated disease treatments, we will present several fundamental and key principles for the design of ROS‐associated nanomedicines. Finally, future perspectives are also discussed in depth in the development of ROS‐associated nanomedicines. It is anticipated that this review article can not only provide a clear venation in ROS‐associated disease therapy, but also present useful strategies and principles for the design of ROS‐associated nanomedicines.

## 2 ROS‐Associated Nanomedicines for Disease Treatments

ROS‐associated nanomedicines include ROS‐downregulating and ROS‐upregulating nanomedicines, which can be used to scavenge and elevate ROS in biological system for antioxidant therapy and ROS‐mediated toxic therapy, respectively. In terms of the nanomedicine‐mediated antioxidant therapy, it is to develop a variety of ROS‐downregulating nanomaterials to scavenge excess ROS for maintaining normal physiological processes. When ROS generation in living organisms increases dramatically, the endogenous antioxidants may fail to scavenge excess ROS, which may induce severe hazard such as the oxidative stress injuries or the migration of cancer cells.^[^ ^24^, ^25^ ^]^ It is well established that one of the most feasible strategies to restrain these ROS‐induced adverse effects is to employ an exogenous ROS scavenger. Currently, many ROS‐scavenging nanomaterials that can effectively relieve aberrant ROS status to stabilize the normal physiological function have been developed. Common ROS‐detoxifying nanoplatforms include carbon‐based nanomaterials (e.g., fullerene (C_60_) and fullerene derivatives) and other inorganic nanomaterials with intrinsic catalytic properties (e.g., platinum (Pt) and CeO_2_).^[^ ^26^, ^27^, ^28^ ^]^ These ROS‐downregulation nanoscavengers exhibit enormous potential in many ROS‐related diseases such as neurodegenerative and inflammatory diseases.

In nanomedicines‐mediated toxic therapy, these nanomedicines can exert the toxic effect of ROS by employing ROS‐upregulating nanoplatforms to enhance ROS generation in pathological sites such as cancer and bacterial infection. The rationale of this approach is that high‐concentration ROS generated by nanosystem can exceed the threshold of endogenous antioxidant system and then result in severe damages of targeted sites. In recent years, aiming at different demands, efforts in ROS‐enhanced nanomedicines have been devoted, in which ROS‐generation nanomedicines can be regarded as ROS delivery platform to upregulate the intracellular redox status to realize site‐specific, deep‐seated, controllable, and oxygen‐independent toxic therapy.^[^ ^29^, ^30^, ^31^, ^32^ ^]^ In general, according to the role of nanomaterials in toxic therapy, ROS‐generation nanomedicines could be roughly divided into two major categories: I) nanomaterials as delivery vehicles to deliver ROS‐generation drugs such as photosensitizers and sonosensitizers, II) nanomaterials themselves as ROS‐generation sources.^[^ ^33^, ^34^, ^35^ ^]^ Common ROS‐enhanced therapeutic modalities include CDT, PDT, SDT, and RT.^[^ ^34^, ^36^, ^37^ ^]^ In order to have a deep insight into the principles for the design of ROS‐regulating nanomedicines, in this section, we will summarize the currently known strategies or approaches in ROS‐associated disease therapy involving antioxidant therapy and ROS‐induced toxic therapy.

### 2.1 ROS‐Scavenging Nanomedicines for Antioxidant Therapy

**Figure 3:**
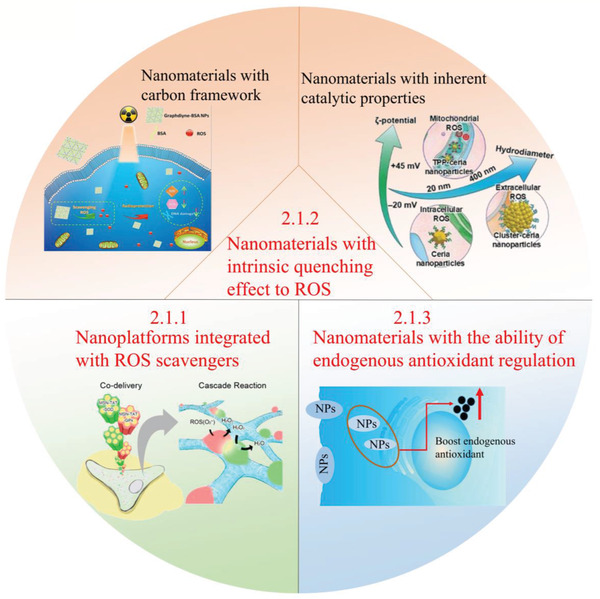
Strategies based on ROS‐scavenging nanomedicines for disease treatments. Effective approaches based on ROS‐scavenging nanomedicines for antioxidant therapy: 1) Nanoplatforms integrated with ROS scavengers. Adapted with permission.^[^ ^53^ ^]^ Copyright 2016, American Chemical Society. 2) Nanomaterials with intrinsic quenching effect to ROS: i) Nanomaterials with carbon framework. Adapted with permission.^[^ ^92^ ^]^ Copyright 2018, American Chemical Society. ii) Nanomaterials with inherent catalytic properties. Adapted with permission.^[^ ^117^ ^]^ Copyright 2018, Wiley‐VCH. 3) Nanomaterials with the ability of endogenous antioxidant regulation.

Excessive ROS produced in biological system can induce oxidative stress, which is closely related to both aging and development of cancer, as well as other diseases such as inflammatory and Alzheimer's disease.^[^ ^38^ ^]^ In general, living organisms consistently maintain the balance between the generation and the elimination of ROS under intracellular antioxidant‐protective systems. However, in the state of oxidative stress, ROS generation increases remarkably, and the endogenous antioxidants do not scavenge all ROS, thereby leading to serious biomolecule damage including DNA, lipids, and proteins.^[^ ^39^ ^]^ It increases the risk of health‐threatening disease. Therefore, in order to prevent or inhibit these oxidative stress injuries, one of the most feasible methods is to employ exogenous antioxidants into biological system. Nevertheless, the traditional antioxidants still face some challenges such as poor stability, high toxicity, and low bioavailability.^[^ ^40^, ^41^, ^42^ ^]^ In recent years, with the development of nanotechnology and nanoscience, novel antioxidant strategies based on multifunctional nanomaterials are widely applied in the construction of ROS scavengers, providing a new opportunity for the development of traditional antioxidant therapy to overcome oxidative stress injuries. Currently, great efforts in ROS‐scavenging nanomedicines have been devoted (**Table** **1**). For ROS‐scavenging designs, nanomaterials as delivery platform of small‐molecule ROS scavengers are usually used to improve the pharmacokinetics of traditional ROS scavengers.^[^ ^43^, ^44^ ^]^ In addition to employing nanomaterials as drug carriers, some nanomaterials with quenching effect to ROS can be directly used as antioxidants, and the ROS‐detoxifying capability mainly attributes to their unique nanostructure or catalytic performance. Furthermore, nanomaterials with the ability of endogenous antioxidant regulation also provide novel approaches to inhibit ROS production.^[^ ^2^ ^]^ To have a deep understanding in the strategies of antioxidant therapy, an overview on the ROS‐downregulating nanomedicines against ROS‐induced injuries will be introduced as follows (**Figure** **3**).

**Table 1:** Summary of the representative ROS‐scavenging nanomedicines

Representative antioxidants	Indications	Active moiety/working mechanism	Refs
Nanoplatforms integrated with ROS scavengers			
Liposomal SOD	Arthritis	SOD converts ^•^O_2_^−^ to H_2_O_2_	^[^ ^48^ ^]^
CAT‐loaded poly(lactic co‐glycolic acid) NPs	Neurodegenerative diseases	CAT converts H_2_O_2_ to H_2_O	^[^ ^303^ ^]^
GPx and SOD‐loaded MSN	ROS‐stressed cells	SOD converts ^•^O_2_^−^ to H_2_O_2_, and then GPx converts H_2_O_2_ to H_2_O	^[^ ^53^ ^]^
GSH‐containing oligomers	H_2_O_2_‐induced oxidative stress in cells	GSH inhibits the toxicity of H_2_O_2_	^[^ ^56^ ^]^
Vitamin C conjugated with silica‐coated Au NPs or lipophilic polyaspartic acid‐based polymer micelles	H_2_O_2_‐induced oxidative stress in cells	ROS‐scavenging efficiency of Vitamin C	^[^ ^59^ ^]^
Curcumin‐loaded PLGA NPs	TBHP (tert‐butyl hydroperoxide)‐induced ROS in cells	ROS‐scavenging efficiency of curcumin	^[^ ^62^ ^]^
Edaravone solid lipid NPs	Noise exposure‐induced ROS in the cochlea	Free radical‐scavenging efficiency of Edaravone	^[^ ^64^ ^]^
*N*‐acetylcysteine‐loaded poly(L‐lactic acid)	Peroxide produced during acute lung injury	Peroxide‐scavenging properties of *N*‐acetylcysteine	^[^ ^61^ ^]^
Coenzyme Q10‐loaded ABC Miktoarm Polymers	ROS‐induced mitochondria damages	Coenzyme Q10 as free radical scavenger	^[^ ^304^ ^]^
Nanomaterials with intrinsic quenching effect to ROS			
Fullerene (C_60_)	Free‐radical‐mediated liver injury	Fullerene can react directly with free radicals, attributing to C_60_’s delocalized *π* double bond system	^[^ ^305^ ^]^
Tris‐malonyl‐C_60_ derivative	^•^O_2_^−^‐induced oxidative damage in mice	Direct radical additions vs catalytic dismutation	^[^ ^74^ ^]^
Fullerenol (C_60_(OH)_24_)	DOX‐induced nephrotoxicity	Antioxidant property of the C_60_ compound	^[^ ^78^ ^]^
Oxidative damage (GO)	X‐ray‐induced oxidative damage in fibroblast cells	Carbon atoms of GO at the edge with higher reactivity allows for efficient capture of oxygen free radicals	^[^ ^86^ ^]^
Graphdiyne NPs	X‐ray‐triggered free radicals in normal cells and tissues	Graphdiyne is consisted of strong *π*‐conjugated structure and highly reactive diacetylenic linkages	^[^ ^92^ ^]^
Citrate‐capped Pt NPs	Stress‐related cerebral cavernous calformation (CCM) disease	Antioxidant nanozyme properties of Pt NPs including POD‐, CAT‐, and SOD‐like antioxidant activities	^[^ ^27^ ^]^
Melanin NPs	*γ*‐ray‐induced oxidative damage in the mouse	Restoration of SOD activity and reduction of MDA in the present of melanin NPs	^[^ ^95^ ^]^
Prussian blue NPs	Injury induced by ROS in some pathological processes	Multienzyme‐like activity including POD, CAT, and SOD activity	^[^ ^96^ ^]^
Mn_3_O_4_ NPs	Oxidative damage induced by ROS in cell	Multienzyme‐like activity including GPx, CAT, and SOD activity	^[^ ^97^ ^]^
NiO NFs	Potential applications in ROS‐related diseases	Redox potential of Ni^II^/Ni^III^ on NiO endows it SOD‐like activity	^[^ ^98^ ^]^
CeO_2_ NPs	H_2_O_2_‐induced oxidative injury model	The mixed valence states of Ce^3+^ and Ce^4+^ on the surface of CeO_2_ NPs endows it enzyme‐mimetics activity	^[^ ^99^ ^]^
Nb_2_C	IR‐induced free radicals against	Nb_2_C with high redox potential and SOD antioxidant enzyme‐mimicking performance	^[^ ^127^ ^]^
Bilirubin NPs	Inflammatory diseases	The ability of bilirubin to scavenge a variety of ROS	^[^ ^129^ ^]^
TPCD	Inflammatory diseases	TPCD with the scavenging ability to a broad spectrum of reactive species	^[^ ^136^ ^]^
Nanomaterials with the ability of endogenous antioxidant regulation			
Chitosan with Se NPs	Intracellular ROS accumulation	Efficiently protecting the activity of GPx and preventing lipofuscin formation	^[^ ^137^ ^]^

#### 2.1.1 Nanoplatforms Integrated with ROS Scavengers

Introducing extracellular small‐molecule ROS scavengers to eliminate excess ROS is a common strategy for maintaining redox homeostasis and reducing oxidative stress injury. Considering that the inherent defects of traditional small‐molecule ROS scavengers, employing nanocarriers may accelerate the further development of small‐molecule antioxidants. Nanomaterials as delivery vehicles not only have the ability to improve the stability and bioavailability of small‐molecule ROS scavengers, but also realize targeted and controlled drug delivery into tissue, cell, or organelles.^[^ ^45^, ^46^ ^]^ Meanwhile, the use of nanocarriers may also decrease the dose of administered drugs, thereby reducing their side effects. Based on the category of the antioxidants in these integrations, these nanomedicines can be divided into nanomaterials integrated with antioxidant enzymes and nanomaterials loaded with non‐enzymatic antioxidants. And the latter one can be subdivided into two classes according to the sources of antioxidants in these compositions, involving endogenous non‐enzymatic antioxidants and exogenous non‐enzymatic antioxidants.

**Figure 4:**
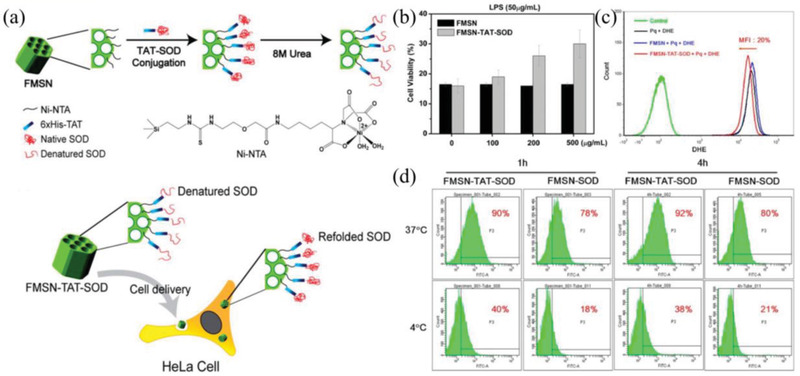
a) Schematic illustration for the synthesis of FMSN‐TAT‐SOD and subsequently provide a protective effect on cells against oxidative stress. b) MTT assay. c) Detection of ROS generation. d) The cellular uptake of NPs determined by FACS analysis. Reproduced with permission.^[^ ^55^ ^]^ Copyright 2013, American Chemical Society.

For nanomaterial integrated with ROS‐detoxifying enzymes, related enzymes mainly include SOD family (convert superoxide anion to H_2_O_2_) and CAT (convert H_2_O_2_ to O_2_). For drug delivery, the key factor is the selection of nanocarriers. And the properties of nanocarriers need to allow a high drug load capacity, excellent biocompatibility, good safety profile, low immunogenicity and toxicity, improved accumulation of drug in targeted sites, as well as the tuneable rate of biodegradation in vivo.^[^ ^47^ ^]^ Therefore, in recent years, some nanomaterials with the above properties such as liposomes, solid lipid NPs, poly (D, L‐lactide co‐glycolide) (PLGA) NPs, and poly (butyl cyanoacrylate) (PBCA) NPs have been applied in the delivery of ROS‐detoxifying enzymes.^[^ ^48^, ^49^, ^50^, ^51^ ^]^ For example, SOD entrapped in long‐circulating liposomes can improve their anti‐inflammatory activity, which attributes to the pegylation‐induced enhancement of circulation half‐lives.^[^ ^48^ ^]^ Furthermore, several drug delivery agents that have potential in penetrating through blood‐brain barrier (BBB) to treat neurologic disorders also deserve some attention, such as biodegradable PBCA and PLGA.^[^ ^51^, ^52^ ^]^ In addition to the above organic nanomaterials, inorganic nanoplatforms such as superparamagnetic iron oxide NPs (SPIONs) and mesoporous silica NPs (MSN) can also be used to deliver antioxidant enzymes.^[^ ^44^, ^53^ ^]^ Here, MSN may be one of the most promising platforms to be used as a multifunctional vehicle because of its good biocompatibility, easy functionalization, uniform pore size, and large surface area.^[^ ^53^, ^54^ ^]^ For example, Chen et al. reported a strategy to enhance the transmembrane delivery of SOD by embedding SOD in MSN (**Figure** **4**).^[^ ^55^ ^]^ In the work, a recombinant gene of human Cu, Zn‐SOD fused with human immunodeficiency virus 1 (HIV) transducing peptide (TAT) was constructed in a bacterial expression vector to overproduce a TAT‐SOD fusion protein, and the TAT‐SOD protein was all in denatured. Then the denatured TAT‐SOD protein was loaded in MSN (FMSN‐TAT‐SOD).^[^ ^55^ ^]^ After translocating in the cell, the denatured SOD loaded on MSN can be refolded to recover activity. From the results of cell viability assay and ^•^O_2_^−^ detection (Figure 4b,c), FMSN‐TAT‐SOD can react with ^•^O_2_^−^ to reduce the level of free radicals, exhibiting protective effect on cells against oxidative stress. Meanwhile, the TAT can enhance cellular uptake and avoid endosome trapping of NPs, which further enhances the antioxidant effect. This strategy provides a novel way to protect enzymatic activity and improves the delivery of antioxidant enzymes.

Besides these ROS‐detoxifying enzymes, common non‐enzymatic antioxidants such as GSH inside the endogenous antioxidant system also play a vital role in ensuring cellular redox homeostasis. GSH, an intracellular thiol, can be found in all tissues, which can react with ^•^O_2_^−^, ^•^OH, ^1^O_2_, and peroxynitrite (ONOO^−^) to avoid oxidative damage from these toxins.^[^ ^56^, ^57^ ^]^ And subsequently, the GSH is gradually translated into the oxidized glutathione (GSSH). The GSSH can be converted back to GSH using nicotinamide adenine dinucleotide phosphate (NADPH) as a reducing cofactor for the donation of electrons, thereby maintaining the ability of intracellular ROS elimination.^[^ ^58^ ^]^ Due to the poor stability and low bioavailability, the GSH always tends to insert with nanomaterials to improve their pharmacokinetics. For example, GSH has been successfully installed in self‐assembled NPs based on poly(ethylene glycol) diacrylate (PEGDA) to protect human brain neuroblastoma cells (SH‐SY5Y) from oxidative stress.^[^ ^56^ ^]^ Among non‐enzymatic antioxidants, some exogenous supplements of antioxidants involving natural and synthetic antioxidants can also be used to strengthen antioxidant defence system, mainly including some small‐molecule ROS scavengers such as vitamin family (e.g., retinoids (vitamin A), ascorbic acid (vitamin C), tocopherol (vitamin E)),^[^ ^59^ ^]^ Coenzyme Q10 (CoQ10),^[^ ^60^ ^]^ *N*‐Acetylcysteine,^[^ ^61^ ^]^ polyphenol antioxidants,^[^ ^62^ ^]^ carotenoids (e.g., lycopene and *β*‐carotene),^[^ ^63^ ^]^ Edaravone (Radicut),^[^ ^64^ ^]^ and NXY‐059 (Cerovive).^[^ ^65^ ^]^ Up to now, the delivery strategies of above non‐enzymatic antioxidants have been widely studied by employing a variety of delivery vehicles such as liposomes, solid lipid NPs, micelles, mesoporous silica, bamboo charcoal NPs, and silica‐coated Au NPs.^[^ ^59^, ^64^, ^66^, ^67^, ^68^, ^69^ ^]^ The presented antioxidants delivery nanoplatforms have distinct advantages in ensuring chemical stability of antioxidants under physiological conditions, providing efficient cellular delivery in a wider concentration range and introducing appropriate release manners. For example, conjugation of vitamin C with Au NPs can realize enhanced cell delivery, which mainly attributes to the covalent conjugation and improved endocytosis.^[^ ^59^ ^]^ In addition, the NPs provide a slow and continuous vitamin C delivery approach based on the ambient glucose concentration. It is well established that to employ nanomaterials for the delivery of ROS scavenger exhibits huge potential in improving therapeutic effect. Nevertheless, the gap in their clinical translation still exists, mainly involving difficulties in biological barrier, repeatability, unknown toxicity, large‐scale production and undesirable biopersistence. Therefore, great efforts on these challenges should be devoted.

#### 2.1.2 Nanomaterials with Intrinsic Quenching Effect to ROS

Nanoplatforms for antioxidants delivery can effectively improve the pharmacokinetics of antioxidants and enhance antioxidant effect. However, the drawbacks of these delivery platforms are obvious, such as premature release of antioxidants and limited dose range by the loading capacity of nanocarriers. And the complexity of synthesis in composited nanomedicines also impedes their application. Therefore, novel antioxidant strategies need to fill in the gaps. In past years, employing NPs with intrinsic quenching effect to ROS have been proposed as a simple but effective strategy to address oxidative stress injury. This quenching effect is mainly attributed to the special framework and inherent catalytic properties of nanomaterials.

##### Nanomaterials with Carbon Framework

One of the most common ROS quenchers may be the nanomaterials with carbon framework such as graphene, graphdiyne, carbon nanotube (CNT), as well as C_60_ and their derivatives.^[^ ^70^, ^71^, ^72^ ^]^ For C_60_ and their derivatives, in the previous report, their antioxidant properties are attributed to the efficacy of the C_60_ compound, which can remove the ROS by the C_60_’s delocalized *π* double bond system.^[^ ^73^, ^74^ ^]^ Nevertheless, some results indicated that C_60_’s free radical‐scavenging performance is not only limited to the direct reaction between the fullerene carbon cage and ROS.^[^ ^73^ ^]^ Although the exact mechanism remains unclear, a number of C_60_ derivatives have been developed and exhibited protective effects in cell culture and animal models of injury.^[^ ^74^, ^75^ ^]^ Among these derivatives, it is well established that water‐soluble fullerene derivatives are attractive and prominent candidates to attenuate oxidative stress, since the hydrophobic essence of original fullerenes is the maximum obstacle in their application to biological systems.^[^ ^26^, ^76^ ^]^ In this regard, one of the most prevailing strategies to obtain water‐soluble fullerene derivatives is to chemically modify fullerenes with functional molecules containing hydrophilic moieties such as —OH, —NH_2_, or —COOH. For example, considerable studies in polyhydroxylated fullerenes (fullerenols) indicate that their water‐soluble and biomedical functions are associated with the number of hydroxyl groups on the fullerene carbon cage.^[^ ^77^ ^]^ These water‐soluble C_60_ derivatives exhibit high electron affinity and radical scavenging activity. And currently, many of them have been used to overcome oxidative stress injuries.^[^ ^78^, ^79^, ^80^ ^]^ For example, a study indicated that fullerenol (C_60_(OH)_24_) plays a protective role in doxorubicin (DOX)‐induced nephrotoxicity through inhibition of oxidative stress.^[^ ^78^ ^]^ Furthermore, other work indicated that C_60_(OH)_24_ can also inhibit the radioactive irradiations‐induced oxidative stress.^[^ ^79^ ^]^

In addition to C_60_ and their derivatives, the carbon nanotube (CNT)‐based nanomaterials can also serve as free radical scavengers. For CNT‐based nanomaterials, they have electron affinity similar to that of C_60_, and their ROS elimination may occur through radical addition to the curved sp^2^‐hybridized carbon nanotube framework.^[^ ^81^, ^82^ ^]^ Many studies have shown that single‐walled carbon nanotubes (SWCNTs) as antioxidants possess strong ROS scavenging ability.^[^ ^72^, ^83^ ^]^ And the functionalization directly affected their antioxidant ability. For example, Lucente‐Schultz et al. investigated the effect of functionalization with the phenolic antioxidant (butylated hydroxytoluene, BHT) on the antioxidant potential of SWCNTs.^[^ ^72^ ^]^ The results showed that BHT can increase the overall antioxidant activity by functionalizing with existing pendant sites on ultrashort SWCNTs. However, when the functionalization of BHT derivative was directly bonded with the SWCNT sidewall via covalent binding, the overall antioxidant activity was gradually decreased with the amount of BHT‐derivative loading, indicating that nanotube itself is an effective free radical scavenger. Similarly, for pristine multi‐walled carbon nanotubes (MWCNTs), their antioxidant ability can be boosted via functionalizing with functional groups.^[^ ^84^, ^85^ ^]^ For example, amino acid‐functionalized MWCNTs exhibit a more robust antioxidant performance than the pristine MWCNTs, which attributes to the significant hydrogen/electron donating activity of amino acid.^[^ ^84^ ^]^ These results indicated that functionalization in CNT has an important effect on their antioxidant activity.

**Figure 5:**
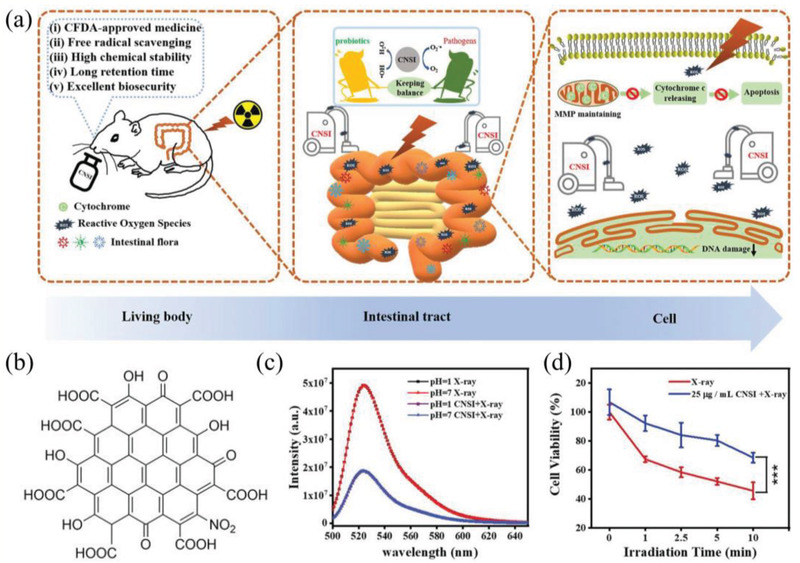
a) CNSI for intestinal radioprotection. b) The chemical structure of CNSI. c) ROS scavenging ability of CNSI in different pH solutions. d) Cell viability of IEC‐6 cells with different treatments. Reproduced with permission.^[^ ^90^ ^]^ Copyright 2020, WILEY‐VCH.

Another noteworthy carbon nanostructures with ROS‐scavenging ability is graphene with unique 2D structures. From the present study, 2D graphene‐based nanomaterials can be used as a new class of antioxidant candidate for free radical scavenging.^[^ ^86^, ^87^, ^88^ ^]^ And few‐layer graphene is more active than monolayer graphene oxide, indicating that the main scavenging sites are closely related with the sp^2^‐carbon network instead of oxygen‐containing functional groups.^[^ ^71^ ^]^ In recent years, graphene‐based nanomaterials exhibit a clear growth potential in reducing injury that is associated with the ROS.^[^ ^84^, ^87^, ^89^ ^]^ For example, Ren et al. synthesized graphene oxide quantum dots (GOQDs) to research the protective effect of GOQDs on 1‐methyl‐4‐phenyl‐pyridinium ion (MPP^+^)‐induced neurotoxicity in P12 cells and larval zebrafish.^[^ ^87^ ^]^ In vitro, the GOQDs can inhibit MPP^+^‐induced ROS generation and SA‐*β*‐Gal expression, etc. In vivo, the GOQDs can also diminish MPP^+^ induced ROS generation and SA‐*β*‐Gal expression, meanwhile, the mortality, malformation rate, apoptosis and mitochondrial damage exhibited significant reduction. The results indicated that GOQDs have the high potential to inhibit neurotoxicity in vitro and in vivo based on its antioxidant activities and metabolic regulation. Although graphene‐based nanomaterials display efficient protective performance, there still exists great difficulties in their clinical transformation. In this regard, conventional drug in new use may be a feasible strategy to rapidly fill in the gap. In recent work, Wang et al. employed carbon NPs suspension injection (CNSI) approved by National Medical Products Administration (NMPA, China) for intestinal radioprotection (**Figure** **5**).^[^ ^90^ ^]^ CNSI is a graphene analog with 12 benzene rings conjugated and carbonylated, which has been applied in lymphatic tracer.^[^ ^91^ ^]^ Owing to its strong delocalized *π*‐conjugated structure, CNSI exhibits enormous potential in radical scavenging. In the work, the results indicated that CNSI can effectively inhibit the apoptosis of the small intestinal epithelial cells and crypt stem cells, and ultimately reduce the damage of the intestine by scavenging X‐ray‐induced ROS.^[^ ^90^ ^]^ Furthermore, CNSI can maintain the balance of the intestinal flora. Because the ROS‐scavenging ability of CNSI can not only decrease the damage of intestinal mechanical barrier to inhibit the large scale proliferation of pathogenic bacteria, but also reduce the effect of X‐ray‐induced ROS on the structure of intestinal flora. The new application of the old nanodrug may provide a new strategy to shorten the clinical conversion time of antioxidant nanomedicines.

Similar to above graphene, graphdiyne also exhibits a promising potential in avoiding ROS‐triggered damage due to its strong delocalized *π*‐conjugated structure and highly reactive diacetylenic linkages.^[^ ^92^, ^93^ ^]^ In recent work, Xie et al. synthesized bovine serum albumin (BSA) modified graphdiyne (GDY‐BSA) NPs to serve as a gastrointestinal radioprotectant.^[^ ^93^ ^]^ First, the GDY NPs possess strong delocalized *π*‐conjugated structure and highly reactive diacetylenic linkages, endowing it with highly efficient radical scavenging activity. Second, the GDY NPs have good chemical stability in the strong acid condition of gastric juice, indicating GDY can ensure its radical scavenging property in gastrointestinal system. Thirdly, small‐sized GDY NPs are able to stay in gastrointestinal tract for a relatively long time and then fully fulfill its drug efficacy. As a result, BSA‐GDY NPs can obviously relieve the X‐ray‐induced damage to gastrointestinal cells via scavenging ROS and inhibiting the ROS‐induced apoptotic signaling pathway. The study in the ROS‐scavenging ability of graphdiyne opens a door for the antioxidant therapy of graphdiyne.

##### Nanomaterials with Inherent Catalytic Properties

With the demand on simple but effective ROS‐scavenging nanomedicines, a large number of nanomaterials with inherent catalytic properties are developed to scavenge ROS. Compared with small‐molecule ROS scavengers, catalytic antioxidants have the advantages of simple manufacturing process, high operability and easy production on a large scale, as well as advanced surface functionalization with stimuli‐sensitive polymers (e.g., pH‐ and H_2_O_2_) and organelle‐directed molecules. Here, we focus on several typical NPs with antioxidant activity including Pt NPs,^[^ ^94^ ^]^ melanin NPs (Me NPs),^[^ ^95^ ^]^ prussian blue NPs (PB NPs),^[^ ^96^ ^]^ manganese oxide (Mn NPs),^[^ ^97^ ^]^ nickel oxide (NiO),^[^ ^98^ ^]^ cerium oxide (CeO_2_),^[^ ^99^ ^]^ and some 2D layered nanomaterials. Many of them possess inherently high SOD‐ or CAT‐like activities.

**Figure 6:**
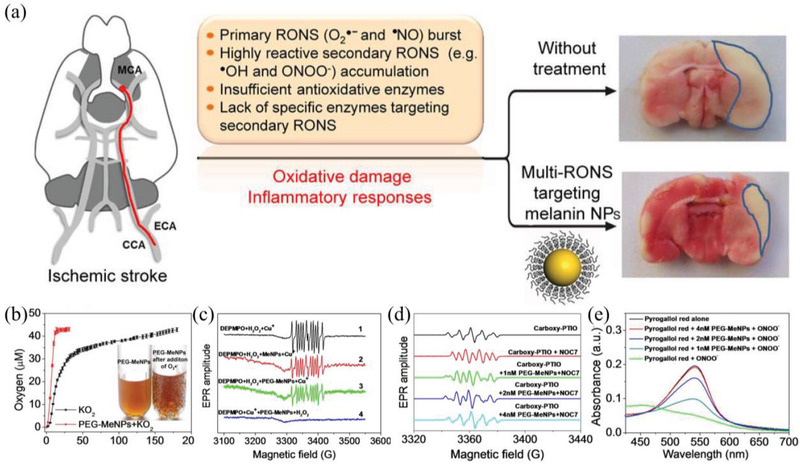
a) Melanin NPs to protect brain from injury in ischemic stroke. b) O_2_ production with or without PEG‐MeNPs. c) EPR spectra of DEPMPO‐OH obtained by trapping ^•^OH. d) The antioxidative activity of PEG‐MeNPs toward ^•^NO. e) ONOO^−^ scavenging effect of PEG‐MeNPs. Reproduced with permission.^[^ ^104^ ^]^ Copyright 2017, American Chemical Society.

Nano‐size noble metal NPs such as Pt, gold (Au) and palladium (Pd) NPs exhibit strong catalytic activity in hydration, hydrogenation, and oxidation reactions, which is mainly attributed to the higher proportion of metal atoms on their surfaces and large specific surface area.^[^ ^100^ ^]^ Such noble metal nanocatalysts are believed to be potential antioxidants. For example, many studies have indicated that Pt NPs possess powerful antioxidant activity, making it a promising candidate as a mimetic SOD/CAT to address the oxidative stress injury.^[^ ^94^, ^101^, ^102^ ^]^ Jawaid et al. reported polyacrylic acid (PAA) capped Pt NPs to suppress radiation‐induced cell apoptosis.^[^ ^94^ ^]^ It mainly attributes to the ROS‐scavenging ability of Pt NPs, which can inhibit the activation of Fas receptor and then decrease the activity of caspase‐8 and caspase‐3, and ultimately reduce the radiation‐induced apoptosis. For Me NPs, it is well known as a potential radical scavenger.^[^ ^95^, ^103^, ^104^ ^]^ Liu et al. revealed its multi‐antioxidative mechanisms, demonstrating the antioxidant potential to reactive oxygen and nitrogen species (RONS) in vitro and in a rat model of ischemic stroke (**Figure** **6**).^[^ ^104^ ^]^ In the work, the synthesized PEG‐Me NPs can effectively scavenge ^•^O_2_^−^ for the transformation of ^•^O_2_^−^ to O_2_, exhibiting SOD‐mimic catalytic activity. Meanwhile, the PEG‐MeNPs can inhibit the generation of ^•^OH by impeding the Fenton reaction, which may be attributed to the strong chelating ability of MeNPs to transition metal ions. In addition, the results demonstrated that PEG‐MeNPs have broad antioxidant activities to resist toxic ^•^NO and ONOO^−^, revealing their radicals‐scavenging potential. For PB NPs, it is an antidote for thallium poisoning, which has been approved by the Food and Drug Administration (FDA, the United States) in 2010.^[^ ^105^ ^]^ In recent years, PB is widely studied in other application such as ROS scavenger.^[^ ^96^, ^106^ ^]^ For example, Zhang et al. demonstrated that PB NPs possess great potential to inhibit or relieve ROS‐induced injury in inflammation model.^[^ ^96^ ^]^ And their ROS scavenging ability is associated with the catalytic activity of three antioxidant enzymes: POD, CAT and SOD.

**Figure 7:**
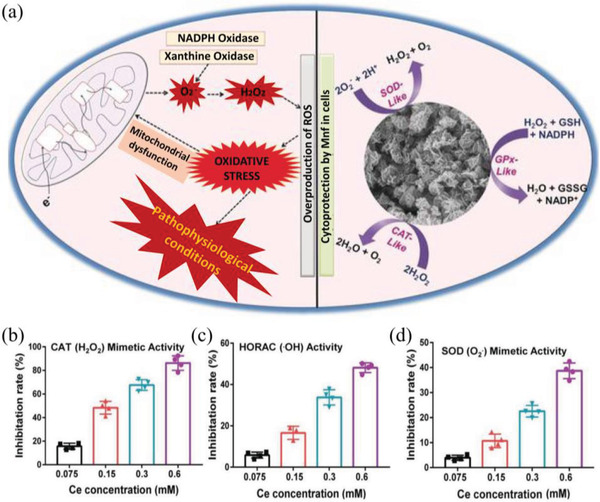
a) A remarkable redox modulatory effect in human cells of Mn_3_O_4_ nanozyme with the catalytic activity of three antioxidant enzymes: CAT, GPx, and SOD. Reproduced with permission.^[^ ^110^ ^]^ Copyright 2017, Wiley‐VCH. b) ROS scavenging activity of ceria NPs mimics catalase (CAT), c) eliminate ^•^OH, and d) SOD. Reproduced with permission.^[^ ^119^ ^]^ Copyright 2019 WILEY‐VCH.

In recent years, metal oxide nanomaterials with specific catalytic properties provide a wide variety of selection for the development of ROS scavengers. One of the most common metal oxide‐based scavengers is the manganese oxide NPs (Mn^4+^). Many studies revealed that Mn^4+^ NPs possess inherent high POD‐, SOD‐, and CAT‐like activities. ^[^ ^107^, ^108^, ^109^ ^]^ Mn NPs (Mn^4+^) can directly catalyze H_2_O_2_ to generate O_2_ and Mn^2+^. Then Mn NPs (Mn^2+^) can mimic the activity of SOD to react with ^•^O_2_^−^ and generate H_2_O_2_. Based on the multienzyme activity of this material, they exhibit a beneficial effect under highly oxidative stress conditions. In addition, Mn‐based nanomaterials with mixed valence states can also exert multienzyme‐like activity for antioxidant therapy. For example, Singh et al. synthesized Mn_3_O_4_ nanoflowers (Mnf) to provide efficient cytoprotection in a Parkinson's disease model, in which the Mnf can mimic three major antioxidant enzymes involving glutathione peroxidase (GPx), CAT, and SOD (**Figure** **7**a).^[^ ^110^ ^]^ In this work, a mechanistic investigation revealed that the fast redox transformations between two valence states (Mn^2+^/Mn^3+^) play a significant role in the multienzyme‐like property of Mnf. Here, the Mn^3+^ can exert CAT and GPx‐like activities, while Mn^2+^ can exhibit SOD activity. In addition, the multienzyme activity of Mnf is size‐ and morphology‐dependent. The results indicated that Mnf as a potential candidate can be considered to address oxidative stress‐induced neurological disorders. Another noteworthy antioxidant metal oxide is NiO NPs. For NiO NPs, the proposed antioxidant mechanism is that the Ni^II^ of NiO NPs as an electron donor can transfer electrons to ^•^O_2_^−^ for the production of H_2_O_2_. And then the Ni^II^ is translated into Ni^III^, in which the Ni^III^ can acquire an electron from ^•^O_2_^−^ to produce O_2_ and Ni^II^.^[^ ^98^ ^]^ The Ni active sites in the NiO NPs as biomimetic SOD provides a promising application to fight ROS‐related diseases. The next well‐known metal oxide as ROS‐scavenging nanozymes is the CeO_2_ NPs. And cerium‐based NPs have become one of the most prevalent ROS scavengers due to the presence of Ce^3+^/Ce^4+^ (oxidized/reduced) and compensating oxygen vacancies, allowing it to abstract or release an electron to neutralize varieties of ROS.^[^ ^28^, ^111^, ^112^, ^113^ ^]^ In general, CeO_2_ NPs have effective redox activity to scavenge H_2_O_2_ and ^•^O_2_^−^, exhibiting promising SOD (Ce^3+^) and CAT (Ce^4+^) mimetic activity to protect cells against oxidative damage.^[^ ^114^, ^115^ ^]^ In recent years, CeO_2_ NPs are widely applied in various ROS‐related diseases such as Parkinson's disease, regenerative wound healing, rheumatoid arthritis, ischemia‐reperfusion injury (IRI), and so on.^[^ ^116^, ^117^, ^118^, ^119^ ^]^ For example, Ni et al. utilize PEGlyated ceria NPs with preferential accumulation in liver to address the hepatic IRI (Figure 7b–d).^[^ ^119^ ^]^ In hepatic IRI, ROS is primarily generated by Kupffer cells or obviously elevated in liver sinusoidal endothelial cells. While PEGlyated ceria NPs trend to locate in the Kupffer and liver sinusoidal endothelial cell, therefore, they are shown to colocalize with ROS in these cells to directly eliminate ROS, thereby suppressing activation of monocyte/macrophage cells and Kupffer cells. And then it significantly minimizes the recruitment and infiltration of neutrophils, as well as reduces the release of proinflammatory cytokines, which can inhibit follow‐up inflammatory reaction in the liver. As a result, ceria NPs exhibited promising protective effect in hepatic IRI. From current results, the progress in the development of nanomaterials with intrinsic catalytic activity for ROS scavenging is slow, and the attention is only paid to several common nanomaterials. In fact, according to the above analysis, many nanomaterials with potential quenching effect to ROS can be used to address ROS‐induced oxidative stress injury. For example, nanomaterials with specific chemical structures such as oxygen vacancies and shifting/mixed valence states may be the feasible candidates for ROS elimination. Here, oxygen vacancies sites or shifting/mixed valence states in the surface of nanomaterials can act as electron traps or donors to effectively and reversibly bind to ROS, resulting in considerable scavenging of ROS. It can be predicted that other NPs mixed with different valence states or integrated with oxygen vacancies may be used as ROS scavenger, especially for H_2_O_2_ and ^•^O_2_^−^.

**Figure 8:**
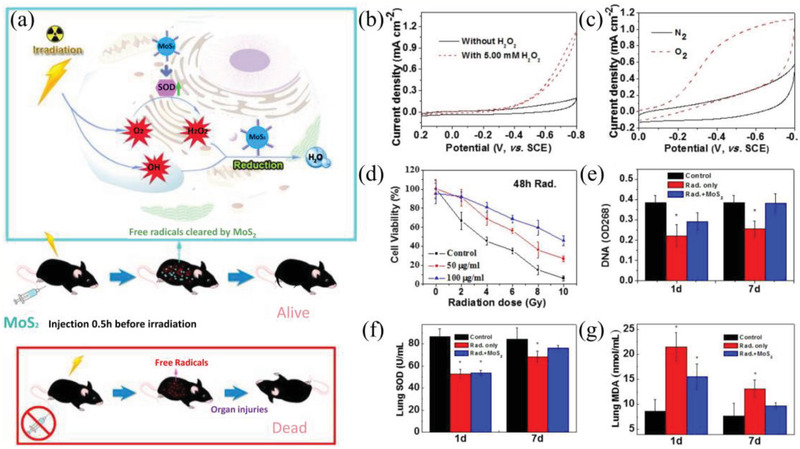
a) Cysteine‐protected MoS_2_ dots with highly catalytic activity as radioprotectants in protection against IR. b) CVs of a glassy carbon electrode (GCE) modified with cysteine‐protected MoS_2_ dots in the presence (dotted) and absence (solid) of 5.00 × 10^−3^ m H_2_O_2_ in N_2_‐saturated 0.01 m pH 7.4 phosphate‐buffered saline (PBS) c) CVs of GCE modified with cysteine‐protected MoS_2_ dots in N_2_‐ (solid) and O_2_‐saturated (dotted) 0.01 m pH 7.4 PBS. d) Radiation dose‐dependent protection in vitro with different injected doses (50 and 100 µg mL^−1^) or without treatment of cysteine‐protected MoS_2_ dots. e) DNA damage of mice 1 and 7 days after treatment with cysteine‐protected MoS_2_ dots. f) SOD levels and g) MDA levels in lung. Reproduced with permission.^[^ ^128^ ^]^ Copyright 2016, American Chemical Society.

In addition, 2D layered nanomaterials also exhibit enormous potential in ROS scavenging. In previous reports, 2D layered materials can be used for hydrogen‐evolution reaction and oxygen‐reduction reactions.^[^ ^120^, ^121^ ^]^ Especially, most of them with ultrasmall size exhibited higher catalytic activity, attributing to their more active edge sites and high specific surface area.^[^ ^122^, ^123^ ^]^ This unique physicochemical property allows them to possess potential advantages in antioxidant applications. Therefore, recently, some ultrasmall or ultrathin 2D nanomaterials such as WSe_2_, Bi_2_Se_3_, WS_2_, MoS_2_, and niobium carbide (MXene) with strong catalytic properties are developed for ROS scavenging in vitro and in vivo.^[^ ^123^, ^124^, ^125^, ^126^, ^127^ ^]^ In a representative work, Zhang et al. designed ultrasmall cysteine‐functionalized MoS_2_ quantum dots (sub‐5 nm) with strong catalytic performance as radioprotectants to investigate their protective effect in ionizing radiation (IR) (**Figure** **8**).^[^ ^128^ ^]^ The electrochemical measurements of cysteine‐functionalized MoS_2_ quantum dots testified their high‐efficiency catalytic activity in H_2_O_2_ and oxygen reduction reactions, resulting in potential removal of ROS. The studies on the mechanism of underlying radioprotection indicated that cysteine‐functionalized MoS_2_ quantum dots can restore the level of SOD and decrease 3,4‐Methylenedioxyamphetamine (MDA) levels of mice by scavenging ROS. Furthermore, MoS_2_ dots can also repair DNA damage and recover some vital biochemical and chemical indicators. As a result, cysteine‐functionalized MoS_2_ quantum dots can effectively decrease IR‐induced damage and increase the surviving fraction. In another work, Ren et al. developed ultrathin 2D niobium carbide MXene (Nb_2_C) to serve as the radioprotectant and also explored its performance in eliminating ROS induced by IR.^[^ ^127^ ^]^ Nb_2_C with SOD‐mimic activity and high redox potential showed a strong scavenging performance against H_2_O_2_, ^•^O_2_^−^, and ^•^OH. The mechanism of Nb_2_C NSs for IR‐triggered ROS elimination is ascribed to the inherent reductive property of Nb_2_C nanosheets. The results of radiation protection in vitro and in vivo indicated that Nb_2_C can effectively prevent IR‐induced damage. It can be seen that 2D layered nanomaterials with ultrasmall size have a promising prospect in antioxidant therapy.

In addition to the above‐mentioned nanoplatforms, recently, some advanced organic materials with ROS‐scavenging ability are assembled into nanoscale particles for the treatments of ROS‐induced disease, such as bilirubin NPs, polydopamine NPs and boronic ester‐derived NPs.^[^ ^129^, ^130^, ^131^, ^132^, ^133^, ^134^, ^135^ ^]^ These organic nanomaterials exhibit improved pharmacokinetics, which can effectively enhance the therapeutic effect. In a representative work, Jon and coworkers synthesized PEGylated bilirubin NPs for anti‐inflammation therapy.^[^ ^129^ ^]^ Bilirubin, a yellow bile pigment, is a high‐efficiency antioxidant that can scavenge various ROS. However, the insolubility in water limit its application. In this work, PEGylated bilirubin can self‐assemble into a nanostructure to improve its intrinsic defect. The results in inflammation model indicated that the bilirubin NPs have strong anti‐inflammatory effects due to their intrinsic ability to effectively scavenge ROS and modulate the immune system. In another report, Zhang et al. prepared ROS‐scavenging material TPCD that is derived from *β*‐cyclodextrin (*β*‐CD) simultaneously functionalized with Tempol (Tpl) and phenylboronic acid pinacol ester (PBAP).^[^ ^136^ ^]^ Here, the Tpl as SOD‐mimetic agents can effectively scavenge ^•^O_2_^−^ and oxygen radicals. The PBAP with catalase‐mimetic activity is able to eliminate H_2_O_2_. The results in this work demonstrated that TPCD can scavenge a broad spectrum of reactive species, which is capable of protecting macrophages from ROS‐induced apoptosis. More importantly, the synthesized TPCD displayed more potent anti‐inflammatory activity in different animal models of inflammatory diseases than the corresponding control small‐molecule drug. This strategy provides new idea to improve the therapeutic effect of small‐molecule antioxidants.

#### 2.1.3 Nanomaterials with the Ability of Endogenous Antioxidant Regulation

Oxidative stress injuries are attributed to the ineffective antioxidant system that fails to maintain redox homeostasis when excessive ROS are produced in cells. In fact, besides to directly introduce exogenous nano‐antioxidants, indirectly promoting the recovering of intracellular antioxidants with the aid of nanotechnology is also a feasible strategy to maintain redox homeostasis. For example, Selenium (Se) acts as a redox center of GPx, therefore, Se supplementation can improve the level of GPx, which can prevent the accumulation of ROS and decrease cell damage.^[^ ^137^ ^]^ Currently, nanomaterials containing selenium have been explored in antioxidant application.^[^ ^138^, ^139^, ^140^ ^]^ Bai et al. synthesized selenium NPs‐loaded chitosan/citrate complex (SeNPs‐C/C), and their antioxidant activities were assessed via employing D‐galactose‐induced aging mice model. The results demonstrated that SeNPs‐C/C is able to boost GPx.^[^ ^138^ ^]^ Furthermore, the activity of SOD and CAT can be also recovered by SeNPs‐C/C, indicating its considerable potential in antioxidant therapy. In addition to Se, research indicated the supplementation with Cu and Zn can be used to increase the activity of Cu, Zn‐SOD.^[^ ^141^ ^]^ Cu and Zn are necessary cofactors of the main antioxidant Cu, Zn‐SOD, which are essential in diet but toxic in excess. However, the safety of metal ion supplementation due to potential danger in free radical generation may be the toughest challenge for their further application. In this regard, some NPs that can slowly release Cu and Zn in biological environment may provide a promising opportunity for the improvement of Cu, Zn‐SOD. The strategy to employ Cu and Zn‐based NPs for Cu, Zn‐SOD elevation needs to be validated in detail. Although nanomaterials‐mediated endogenous antioxidant regulation may not be mature enough for application, it provides a potential direction to seek novel antioxidants to attenuate oxidative stress injuries.

### 2.2 ROS‐Enhanced Nanomedicines for ROS‐Induced Toxic Therapy

**Figure 9:**
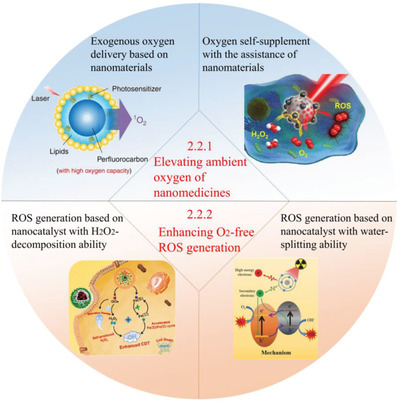
Strategies based on ROS‐enhanced nanomedicines for ROS‐induced toxic therapy. 1) Elevating ambient oxygen of nanomedicines: i) Exogenous oxygen delivery based on nanomaterials. Adapted with permission.^[^ ^150^ ^]^ Copyright 2015, Springer Nature. ii) Oxygen self‐supplement with the assistance of nanomaterials. Adapted with permission.^[^ ^181^ ^]^ Copyright 2017, American Chemical Society. (2) Enhancing O_2_‐free ROS generation: i) ROS generation based on nanocatalyst with H_2_O_2_‐decomposition ability. Adapted with permission.^[^ ^196^ ^]^ Copyright 2018, American Chemical Society. ii) ROS generation based on nanocatalyst with water‐splitting ability. Adapted with permission.^[^ ^225^ ^]^ Copyright 2017, WILEY‐VCH.

ROS effect is a double‐edged sword. A proper concentration of ROS can be used as a messenger to mediate normal physiological processes. Nevertheless, excess ROS are capable of destroying the antioxidant ability of cells and then inducing cell death. In terms of the excessive ROS, its killing effect may be an advantage in other disease treatments such as antineoplastic and antimicrobial therapy. Therefore, in the last few years, many approaches by employing ROS‐enhanced nanomedicines to raise the cellular redox have been becoming a hotspot. Biologically relevant ROS mainly include H_2_O_2_, ^•^O_2_^−^, ^•^OH, ^1^O_2_, etc., and many present strategies focus on the elevation of these ROS with the aid of nanomaterials. These emerging ROS‐elevated nanomaterials with different physicochemical features display varied functions in antineoplastic and antimicrobial therapy. Common ROS‐generated nanoplatforms are widely applied in PDT, RT, or SDT.^[^ ^142^, ^143^, ^144^ ^]^ However, most of the ROS‐generated processes are high dependence on the ambient O_2_, such as ^1^O_2_ and ^•^O_2_^−^. Entirely depending on the endogenous O_2_ supply is not conducive to the effective and persistent production of ROS in some pathological sites such as tumor. It is unable to rescue the O_2_ supply because the incomplete vasculature would result in the low efficiency of blood circulation in tumor. In order to optimize the performance of ROS generation of nanomedicines, a deep insight into the mechanism of O_2_‐associated ROS generation could provide essential information for the development of ROS‐elevated strategies, and open up new possibilities to improve the therapeutic effect of disease. In the preceding sections, we have reviewed various ROS‐downregulating strategies for antioxidant therapy mediated by nanomaterials. And here, from the perspective of O_2_ roles, an overview on the nanomedicine‐mediated, ROS‐elevated therapeutic strategies against cancer will be provided (**Figure** **9**). And some representative ROS‐enhanced nanomedicines are shown in **Table** **2**.

**Table 2:** Summary of the representative ROS‐enhanced nanomedicines

Representative ROS‐elevated nanomedicines	Therapeutic modalities	ROS types	Working mechanism	Strategies	Refs
Elevating ambient oxygen of nanomedicines					
PFC and IR780‐coencapsulated lipids NPs	PDT	^1^O_2_	PFC can improve the ambient oxygen photosensitizer IR780, and then accelerate generation of ^1^O_2_ to enhance photodynamic effect	Exogenous oxygen delivery	^[^ ^150^ ^]^
Hb conjugated polymeric micelles	PDT	^1^O_2_	Hb with oxygen‐binding capacity can enhance the ^1^O_2_ generation of photosensitizer zinc phthalocyanine	Exogenous oxygen delivery	^[^ ^159^ ^]^
human serum albumin (HSA)‐stabilized PFC nanodroplets	PDT and RT	^1^O_2_	PFC nanodroplets can adsorb oxygen in the lung and rapidly release oxygen in the tumor under US, which enhance PDT and RT	Exogenous oxygen delivery; US‐triggered rapid oxygen release	^[^ ^153^ ^]^
MOF (UiO‐66) conjugated with indocyanine green (ICG)	PDT	^1^O_2_	Photothermal property of ICG could facilitate the burst release of O_2_, which significantly improve the PDT effects of ICG	Exogenous oxygen delivery; NIR‐induced oxygen burst release	^[^ ^149^ ^]^
ATO/VER NPs	PDT	^1^O_2_	ATO can reduce cellular oxygen consumption by inhibition of mitochondria respiratory chain, and then enhance VER to generate ^1^O_2_ in hypoxic tumor.	Oxygen elevation by inhibiting cellular oxygen consumption	^[^ ^173^ ^]^
MnFe_2_O_4_ NPs‐anchored MSN	PDT	^1^O_2_	MnFe_2_O_4_ NPs catalyze H_2_O_2_ tumor microenvironment O_2_ generation, and then MFNs loaded with Ce6 under continuous oxygen supply can enhance ROS generation	In situ oxygen generation by decomposing cellular H_2_O_2_	^[^ ^181^ ^]^
MnO_2_‐Ce6 NPs	PDT	^1^O_2_	MnO_2_ NPs with high reactivity toward H_2_O_2_ can increase O_2_ generation in tumor, and then promote ^1^O_2_ generation and enhance PDT effects	In situ oxygen generation by decomposing cellular H_2_O_2_	^[^ ^306^ ^]^
Carbon‐dot‐decorated C_3_N_4_ nanocomposite (CNN)	PDT	^1^O_2_	A 630 nm laser was used to trigger CCN to split water to generate O_2_, meanwhile, 630 nm laser irradiation can activate the photosensitizer PpIX on CNN for ^1^O_2_ generation	Photocatalyst for splitting water to generate O_2_	^[^ ^187^ ^]^
Ultrathin graphdiyne oxide (GDYO) nanosheets	PDT	^1^O_2_	GDYO under 660 nm laser irradiation are able to efficiently catalyze water oxidation to release O_2_ and induce blood perfusion, promoting ^1^O_2_ generation	Photocatalyst for splitting water to generate O_2_	^[^ ^188^ ^]^
Enhancing O_2_‐free ROS generation					
Fe meta‐organic framework	Fenton cancer therapy	^•^OH	Iron present on the rMOF‐FA can release into solution, reacting with high levels of H_2_O_2_ to generate ^•^OH	Catalyzing H_2_O_2_ for O_2_‐free ROS generation	^[^ ^307^ ^]^
GOD‐Fe_3_O_4_@DMSNs nanocatalysts	Fenton cancer therapy	^•^OH	GOD catalyze the glucose into abundant H_2_O_2_ in tumor region, and then the elevated H_2_O_2_ is catalyzed by the downstream Fe_3_O_4_ NPs	GOD for H_2_O_2_ elevation; catalyzing H_2_O_2_ for O_2_‐free ROS generation	^[^ ^195^ ^]^
Fe_3_O_4_@MSN encapsulating doxorubicin (DOX)	Fenton cancer therapy + chemotherapy	^•^OH	DOX can activate nicotinamide adenine dinucleotide phosphate oxidases (NOXs) for H_2_O_2_ elevation, and then the elevated H_2_O_2_ is catalyzed by the downstream Fe_3_O_4_ NPs	DOX for H_2_O_2_ elevation; catalyzing H_2_O_2_ for O_2_‐free ROS generation	^[^ ^308^ ^]^
Fe_3_O_4_ NPs loading cisplatin(IV) prodrugs	Fenton cancer therapy + chemotherapy	^•^OH	Cisplatin(IV) prodrugs can be activated by intracellular GSH, then Cisplatin(II) activate NOXs for H_2_O_2_ elevation, and then the elevated H_2_O_2_ is catalyzed by the downstream Fe_3_O_4_ NPs	GSH consumption; cisplatin for H_2_O_2_ elevation; Catalyzing H_2_O_2_ for O_2_‐free ROS generation	^[^ ^309^ ^]^
MnO_2_‐coated MSN NPs	Fenton cancer therapy	^•^OH	MnO_2_ shell can react with GSH to yield Mn^2+^, and then Mn^2+^‐trigger ^•^OH production from H_2_O_2_	GSH depletion; catalyzing H_2_O_2_ for O_2_‐free ROS generation	^[^ ^194^ ^]^
UCNPs@silica core‐shell NPs loaded with Fe^2+^ ion	Photo‐Fenton cancer therapy	^•^OH	UCNP cores can convert NIR light to UV or visible photons to catalyze photo‐Fenton reaction	Near infrared‐assisted Fenton reaction	^[^ ^201^ ^]^
Cu_2−_ *_x_*Se NPs	Photo‐Fenton cancer therapy	^•^OH	NIR‐II irradiation can promote the conversion of Cu^2+^ and Cu^+^	X‐ray‐driven Fenton reaction	^[^ ^189^ ^]^
Cu_2_(OH)PO_4_ nanocrystals	RT	^•^OH	X‐ray can trigger Cu^I^ sites generation on Cu_2_(OH)PO_4_ nanocrystals, serving as a catalyst to efficiently decomposing overexpressed H_2_O_2_ in the tumor	X‐ray‐induced Fenton reaction	^[^ ^202^ ^]^
Au–Bi_2_S_3_ NPs	RT	^•^OH	Schottky barrier in Au–Bi2S3 can remarkably improve the utilization of a large number of X‐ray‐induced low energy electrons for H_2_O_2_ decomposition	X‐ray‐induced H_2_O_2_ decomposition	^[^ ^204^ ^]^
Reduced graphene oxide (rGO) coupled with BiP_5_W_30_	RT	^•^OH	rGO can BiP_5_W_30_ NPs can improve radiocatalytic activity through promoting e^−^–h^+^ separation to decomposing H_2_O_2_ into ^•^OH. In addition, BiP_5_W_30_ NPs can deplete GSH to further enhance ^•^OH generation	X‐ray‐induced H_2_O_2_ decomposition; GSH depletion	^[^ ^205^ ^]^
TiO_2_‐coated UCNPS	PDT	^•^OH,H^+^, ^•^O_2_^−^	UCNPs can efficiently convert NIR light to UV emission, then activate TiO_2_ for the formation of an e^−^–h^+^ pair and generation of intracellular ROS	NIR‐induced deep tissue penetration; catalyzing H_2_O for O_2_‐free ROS generation	^[^ ^32^ ^]^
SrAl_2_O_4_:Eu^2+^@MC540	RT+PDT	^1^O_2_	Scintillator emits numerous photons of low energy that can trigger MC540 for ^1^O_2_ generation	X‐ray trigger deep PDT	^[^ ^30^ ^]^
Ce^III^‐doped LiYF_4_@SiO_2_@ZnO nanostructure	RT	^•^OH, ^•^O_2_^−^	Scintillator emits numerous photons of low energy that can trigger ZnO for the formation of an e^−^–h^+^ pair and free radicals	X‐ray‐induced deep tissue penetration; catalyzing H_2_O for O_2_‐free ROS generation	^[^ ^222^ ^]^
LiLuF_4_:Ce@SiO_2_@Ag_3_PO4@Pt(IV)	RT+ chemotherapy	^•^OH, ^•^O_2_^−^	Scintillator emits numerous photons of low energy that can trigger Ag_3_PO_4_ for the formation of an e^−^–h^+^ pair and free radicals. Meanwhile, cisplatin(IV) prodrugs as sacrificial agent can increase the yield of free radicals, thereby exerting chemotherapy effect	X‐ray‐induced deep tissue penetration; inhibiting e^−^–h^+^ pair recombination; catalyzing H_2_O for O_2_‐free ROS generation	^[^ ^223^ ^]^
Bi_2_WO_6_ nanoplates	RT	^•^OH, ^•^O_2_^−^	Under X‐ray irradiation, Bi_2_WO_6_ generate e^−^–h^+^ pair and subsequently promoting the generation of ROS	X‐ray‐induced deep tissue penetration; catalyzing H_2_O for O_2_‐free ROS generation	^[^ ^224^ ^]^
BiOI@Bi_2_S_3_ heterojunction NPs	RT	^•^OH, ^•^O_2_^−^	BiOI@Bi_2_S_3_ NPs inhibit rapid recombination of e^−^–h^+^ pair to promoting the generation of ROS under X‐ray irradiation	X‐ray‐induced deep tissue penetration; catalyzing H_2_O for O_2_‐free ROS generation	^[^ ^225^ ^]^
Au‐TiO_2_ nanocomposite	SDT	^•^OH, ^•^O_2_^−^	Au‐TiO_2_ nanocomposite can increase ROS generation by enhancing the energy absorption and reducing the e^−^–h^+^ pair recombination	US‐induced deep tissue penetration; inhibiting e^−^–h^+^ pair recombination; catalyzing H_2_O for O_2_‐free ROS generation	^[^ ^228^ ^]^
MnWO*_X_* NPs	SDT	^•^OH, ^1^O_2_	MnWO*_X_* NPs can reduce the e^−^–h^+^ pair recombination for enhanced ROS generation and deplete intracellular GSH	US‐induced deep tissue penetration; inhibiting e^−^–h^+^ pair recombination; catalyzing H_2_O for O_2_‐free ROS generation	^[^ ^229^ ^]^

#### 2.2.1 Elevating Ambient Oxygen of Nanomedicines

In traditional ROS‐related therapy such as PDT, SDT, and RT, the content of ambient O_2_ determines the level of ROS production. Considering the critical roles of O_2_ in ROS generation, directly infusing exogenous O_2_ into pathological tissue to enhance ambient oxygen of nanomedicines may be an attractive strategy to achieve highly efficient therapy. In previous studies, pure oxygen can be provided to patient in a pressurized sealed chamber and then facilitate oxygen transport to the hypoxic tumor for a hyperbaric oxygen (HBO) therapy.^[^ ^145^ ^]^ Unfortunately, side effects such as hyperoxic seizures and barotrauma severely limit its development in clinic.^[^ ^146^ ^]^ In recent years, some innovative O_2_‐delivery or O_2_‐generation strategies based on nanomaterials are developed for high‐efficiency ROS generation, including exogenous oxygen delivery and oxygen self‐supplement to pathological tissue.

##### Exogenous Oxygen Delivery Based on Nanomaterials

In terms of the O_2_ delivery system, the key factor is the O_2_ carrier. Common O_2_ carriers include perfluorocarbon (PFC), hemoglobin (Hb), and metal‐organic frameworks (MOFs).^[^ ^147^, ^148^, ^149^ ^]^ For O_2_ carriers, the stability, circulation time and penetrability are crucial for their biological application. Therefore, in recent years, to optimize the O_2_‐delivering performance of O_2_ carriers, nanomaterials and O_2_ carriers are often combined to construct new O_2_ delivery system. For example, several PFC‐coupled NPs have been designed to increase the therapeutic outcome of PDT, RT, and SDT via improving the O_2_ level.^[^ ^150^, ^151^, ^152^ ^]^ A typical work by Cheng et al. reported a composite nanosystem by coating photosensitizer IR780 and PFC with lipid monolayer for PFC‐mediated PDT.^[^ ^150^ ^]^ The inner PFC core is capable of enriching oxygen to accelerate ^1^O_2_ generation, resulting in enhanced tumor inhibition. In common O_2_ reservoir based on PFC, O_2_ is provided in the form of slow release. However, in recent years, a burst O_2_ release and diffusion is pursued because the rapid release behavior can realize the maximization of O_2_ concentration in a short time. The high‐concentration O_2_ can speed up the ROS generation from the perspective of reaction kinetics. Fortunately, some nanoplatforms that can promote O_2_ release by harvesting exogenous stimuli have been fabricated, such as albumin‐stabilized PFC nanodroplets under low‐frequency ultrasound (US) for PDT and RT,^[^ ^153^ ^]^ PFC‐loaded Bi_2_Se_3_ NPs with near‐infrared (NIR) irradiation for RT,^[^ ^154^ ^]^ and polymer‐based PFC nanovesicles in response to US for SDT.^[^ ^155^ ^]^

**Figure 10:**
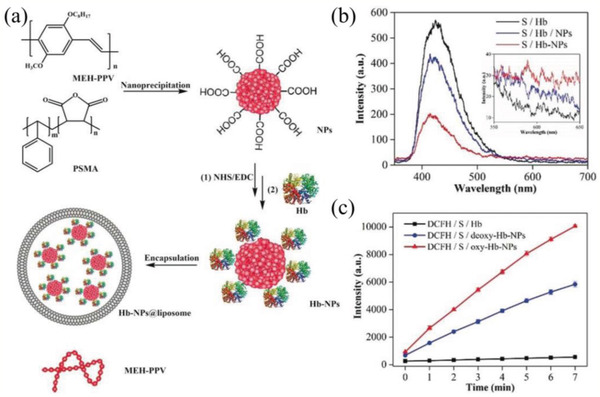
a) Schematic illustration of the preparation of Hb‐NPs@liposome. b) Luminescence spectra of luminol in the presence of Hb, a mixture of Hb and NPs, and Hb‐NPs; inset shows the enlarged view of the fluorescence intensity of MEH‐PPV NPs. c) Evaluation of ROS yield. Reproduced with permission.^[^ ^160^ ^]^ Copyright 2019, Wiley‐VCH.

Hb is another representative oxygen carriers, which can serve as blood substitutes to bind and transfer oxygen for re‐establishing oxygen level in tissues.^[^ ^156^ ^]^ Nevertheless, stroma‐free Hb shows adverse impact, such as low stability, short circulation time and renal toxicity.^[^ ^157^, ^158^ ^]^ In this regard, Hb integrating with nanocarriers such as micelles and vesicles can overcome these shortcomings.^[^ ^158^, ^159^ ^]^ Meanwhile, the nanosized carriers allow Hb to more easily permeate through tumor vasculature, realizing homogeneous delivery of O_2_ in tumor. Recently, Hb‐loaded NPs that hold an effective oxygen supply capacity have exhibited considerable advantage in assisting ROS production for ROS‐enhanced therapy. For example, Jiang et al. fabricated Hb‐linked conjugated polymer NPs that were encapsulated in fusogenic liposomes (Hb–NPs@liposome) to realize oxygen supply and self‐luminescing (**Figure** **10**).^[^ ^160^ ^]^ When Hb–NPs@liposome are internalized by tumor cells, Hb is as the catalyst of the chemiluminescence system for the activation of luminol in the presence of H_2_O_2_, the NPs with polymer poly[2‐methoxy‐5‐(2‐ethylhexyloxy)‐1,4‐phenylenevinylene] (MEH‐PPV) can absorb the chemiluminescence of luminol via chemiluminescence resonance energy transfer (CRET), and then facilitate ROS generation by sensitizing the O_2_ supplied by Hb to kill tumor cells. Another type of widely explored oxygen carriers is metal‐organic frameworks (MOFs), which is an emerging class of crystalline materials comprised of inorganic metal ion nodes bound via organic linkers.^[^ ^161^, ^162^ ^]^ Owing to their unique mesoporous structure and large surface area, MOFs have been widely applied for gas storage. Recently, several MOFs have exhibited promise as sorbent materials for storing oxygen.^[^ ^149^, ^161^, ^163^ ^]^ For example, zirconium (IV)‐based MOF (UiO‐66) can act as a carrier for oxygen storage. Here, UiO‐66 combined with indocyanine green (ICG) by coordination reaction, and then the as‐prepared NPs were encapsulated inside red blood cell (RBC) membranes.^[^ ^149^ ^]^ Subsequently, the ICG can generate initial ^1^O_2_ with 808 nm laser irradiation to decompose RBC membranes. Meanwhile, the photothermal effect of ICG can promote the rapid release of O_2_ from UiO‐66. The released O_2_ can markedly enhance the ^1^O_2_ generation and amplify PDT effect for hypoxic tumor therapy.

##### Oxygen Self‐Supplement with the Assistance of Nanomaterials

**Figure 11:**
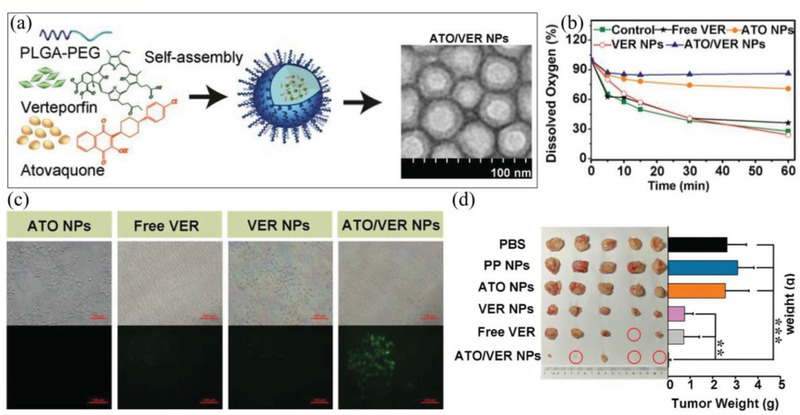
a) Schematic illustration of the preparation of the ATO/VER NPs and representative TEM images of the ATO/VER NPs. b) Determination of 4T1 cell oxygen consumption by measurement of DO content 4 h after administration without laser irradiation. The initial value was taken to be 100%. c) Determination of intracellular ROS generation (green fluorescence) by an inverted fluorescence microscope. d) Representative images of the excised tumors after different treatments. Reproduced with permission.^[^ ^173^ ^]^ Copyright 2019, WILEY‐VCH.

Exogenous oxygen delivery is capable of improving the O_2_ level of pathological sites and then enhancing the ROS generation to improve killing effect. However, premature release of O_2_, transient generation of O_2_, the limited O_2_ capacity and poor penetration of O_2_ gas hamper their clinical application. Therefore, some innovative strategies have been proposed to proceed oxygen self‐supplement, mainly including nanomaterials‐triggered blood perfusion enhancement, tumor vascular normalization and inhibition of cell respiratory chain. In tumor therapy, the intratumoral blood perfusion is relatively low due to the tortuous and leaky blood vessels, and the fast‐growing tumor cells induce the rapid consummation of oxygen, leading to an inefficient oxygen supply.^[^ ^164^ ^]^ The inefficient oxygen supply to the tumor has resulted in strong resistance of ROS‐mediated therapeutic modalities such as PDT or RT for many types of cancers. In past studies, it was found that tumor temperature elevation to a mild temperature (40–42 °C) can enhance the tumor oxygenation due to an increase in blood flow.^[^ ^37^, ^165^ ^]^ Encouraged by the outstanding photothermal effect of some nanomaterials under light irradiation, many studies in tumor therapy utilize these nanomaterials as heat generators to elevate O_2_ level of tumor by enhancing blood perfusion. This strategy exhibits a promising application prospect in RT.^[^ ^144^, ^166^, ^167^, ^168^ ^]^ From current results in RT, heat can indeed enhance blood perfusion to trigger tumor oxygenation, thereby enhancing radiotherapeutic efficacy. Nevertheless, the effect of blood perfusion on ROS generation in RT should be evaluated in detail because the role of oxygen in RT is relatively complicated.^[^ ^169^, ^170^ ^]^ Furthermore, the blood perfusion induced by heat is transitory, therefore, the appropriate therapeutic window needs to be explored for maximizing the supplementary role of blood perfusion. In addition to the blood perfusion enhancement, transient vascular normalization is also of great significance to guarantee the O_2_ supply via strengthening blood vessel integrity and then increasing blood‐flow perfusion.^[^ ^171^, ^172^ ^]^ The tumor vascular normalization theory provides a potential opportunity to open the door for the rational use of antiangiogenic agents in ROS‐induced toxic treatments. Another novel strategy for elevating O_2_ is to reduce the oxygen consumption rate by perturbing the normal energy metabolism process. In recent work, Fan et al. reported a unique oxygen‐regulating strategy for O_2_ elevation of tumor region, thus to promote ROS generation and enhance the efficiency of PDT (**Figure** **11**).^[^ ^173^ ^]^ In detail, dual‐drug NPs (ATO/VER/PLGA‐PEG NPs) containing photosensitizer verteporfin (VER) and oxygen‐regulator atovaquone (ATO) can efficiently deliver VER and ATO into tumor. Here, ATO can inhibit mitochondria respiratory chain to reduce cellular oxygen consumption, and then facilitate VER to produce a better number of ^1^O_2_ in hypoxic cancer cells. As a result, the dual‐drug NPs showed strong PDT effects both in vitro and in vivo. Although the aforementioned strategies of oxygen self‐supplement including blood perfusion enhancement, transient vascular normalization and the inhibition of oxygen consumption to enhance ROS generation are still far away from clinical application, they offer beneficial ideas for O_2_ elevation and ROS generation.

**Figure 12:**
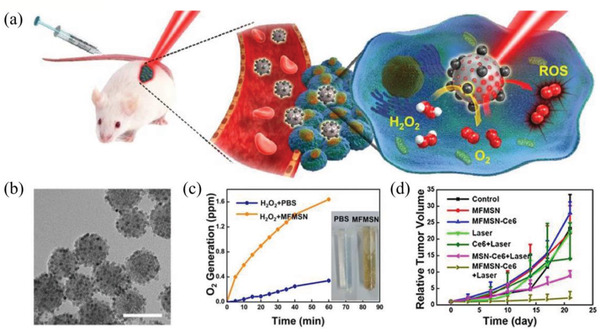
a) Schematic illustration of MFMSNs. b) TEM image of MFMSNs. Scale bar, 60 nm. c) O_2_ generation after treating with MFMSNs in PBS. d) Tumor volume changes. Reproduced with permission.^[^ ^181^ ^]^ Copyright 2017, American Chemical Society.

Besides above strategies, in recent years, employing nanomaterials that can induce catalytic reaction inside tumor for O_2_ generation in situ has become a promising approach to realize oxygen self‐supplement and enhance ROS generation. Considering the presence of overexpressed H_2_O_2_ in tumor, a common way for in situ O_2_ generation is to decompose endogenous H_2_O_2_ with the aid of nanomaterials.^[^ ^174^, ^175^, ^176^, ^177^, ^178^ ^]^ These O_2_‐evolving nanomaterials can catalyze H_2_O_2_ and then locally generate O_2_, ultimately realizing tumor reoxygenation to enhance ROS‐induced toxic therapy. One of the most widely studied strategies in decomposing H_2_O_2_ into O_2_ is to insert CAT into nanomaterials.^[^ ^175^, ^179^, ^180^ ^]^ For example, Chen et al. reported the O_2_‐evolving PDT NPs consisting of PLGA shell and methylene blue (MB)/CAT core.^[^ ^179^ ^]^ The endogenous H_2_O_2_ can penetrate the shell of NPs into the core and subsequently be decomposed into O_2_ by CAT, resulting in a broken shell and the photosensitizer MB release. The released MB under irradiation can produce a large number of highly toxic ^1^O_2_ with the aid of generated O_2_ to kill cancer cells. In addition, some nanomaterials with CAT‐like activity such as MnFe_2_O_4_ NPs, MnO_2_ NPs, and CeO_2_, as well as Pt and Au nanomaterials have also been used to selectively improve the O_2_ level of cancer.^[^ ^181^, ^182^, ^183^, ^184^, ^185^, ^186^ ^]^ For example, Kim et al. designed MnFe_2_O_4_ NPs to anchor in mesoporous silica NPs (MFMSNs) loaded with the photosensitizer molecule chlorin e6 (Ce6) for oxygen self‐supplement (**Figure** **12**).^[^ ^181^ ^]^ MnFe_2_O_4_ NPs can effectively catalyze H_2_O_2_ and allow continuous O_2_ production at a small amount of the NPs for enhanced PDT. It can be seen that H_2_O_2_‐triggered O_2_ elevation provides a feasible strategy to overcome hypoxia for effectively ROS‐mediated killing of cancer cells.

**Figure 13:**
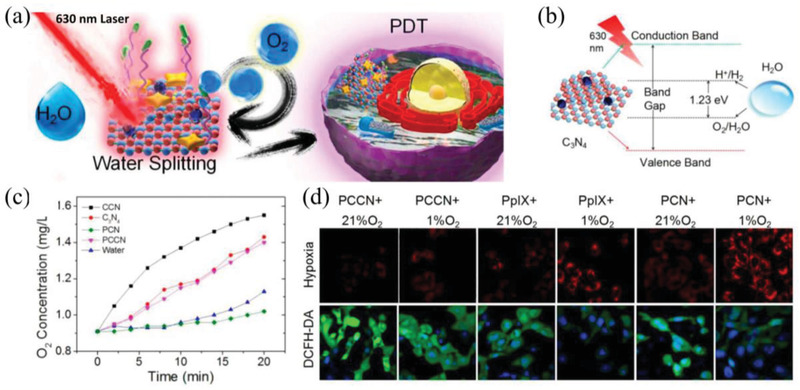
a) Schematic diagram of 630 nm light‐driven water splitting enhanced PDT. b) Schematic illustration of the C_3_N_4_‐mediated water splitting process. c) O_2_ generation curve. d) CLSM images of PDT‐induced hypoxia reversion and intracellular ROS generation. Reproduced with permission.^[^ ^187^ ^]^ Copyright 2016, American Chemical Society.

Despite huge advantage, the intrinsically limited H_2_O_2_ level in tumor cell dramatically restricts the O_2_ generation yield in H_2_O_2_‐triggered O_2_ elevation and thus may reach only moderate efficacy in ROS‐induced therapy. In recent years, photocatalytic nanomaterials with water‐splitting ability, that can locally produce O_2_ in tumor by decomposing the H_2_O molecule in biological tissues under light irradiation, are more appealing in overcoming low‐level O_2_ of tumor because water is the most abundant molecule in cells. For example, a carbon‐dot‐decorated C_3_N_4_ nanocomposite (CCN) was used to trigger water splitting to generate O_2_ (**Figure** **13**).^[^ ^187^ ^]^ Here, carbon dots were used to reduce the band gap of C_3_N_4_, resulting in water splitting that can be triggered by red light. The C_3_N_4_‐mediated water splitting mechanism meets the requirements: first, the band gap of C_3_N_4_ can be activated under light irradiation to form electron–hole (e^−^–h^+^) pairs; second, the conduction band (CB) and valence band (VB) can match with the reduction (H^+^/H_2_) and oxidation potential (H_2_O/O_2_) of water (Figure 13b). And then, the photosensitizer protoporphyrin IX (PpIX) in CCN with 630 nm laser irradiation can transform O_2_ into cytotoxic ^1^O_2_ for tumor therapy. In another work, ultrathin graphdiyne oxide (GDYO) nanosheets can also trigger sufficient O_2_ under infrared irradiation (660 nm).^[^ ^188^ ^]^ Currently, only a few water‐splitting nanomaterials have been applied for tumor reoxygenation for ROS‐triggered cancer treatment. It primarily lies in the difficulties of developing the high‐efficient photocatalysts and exploring appropriate exciting light with better penetration of biological tissues. Here, in terms of the penetration depth, upconversion NPs (UCNPs) that can convert NIR light into ultraviolet/visible (UV/Vis) region emissions or scintillants that can convert X‐ray into UV/Vis region emissions combining with water‐splitting nanomaterials may be used to trigger O_2_ generation for ROS promotion in deep sites.

#### 2.2.2 Enhancing O_2_‐Free ROS Generation

In the aforementioned sections, we have introduced various O_2_‐upregulating strategies assisted by nanomaterials for ROS‐induced toxic therapy. Nevertheless, the delivery or chemical production of O_2_ in pathological tissue remain many challenges for further clinical application, such as seeking continuous productivity of O_2_ or expanding the therapeutic window. Therefore, some emerging strategies have been developed to reduce or avoid the dependence of nanomaterials‐mediated ROS generation on O_2_. These strategies can directly promote O_2_‐free ROS production by decomposing intracellular H_2_O_2_ or H_2_O base on the catalytic performance of nanomaterials, ultimately resulting in damages of pathological cells.

##### ROS Generation Based on Nanocatalyst with H_2_O_2_‐Decomposition Ability

**Figure 14:**
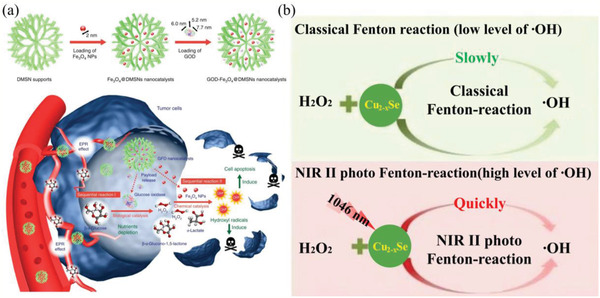
a) Fabrication and catalytic‐therapeutic schematics of sequential GFD NCs. Reproduced with permission.^[^ ^195^ ^]^ Copyright 2017, Springer Nature. b) Schematic comparison of the classical Fenton reaction and the NIR‐II photo‐Fenton reaction. Reproduced with permission.^[^ ^189^ ^]^ Copyright 2019, WILEY‐VCH.

The unique biochemical feature of TME with elevated H_2_O_2_ level provides the possibility to precisely discriminate tumor and normal tissues during therapy. In recent years, a number of studies have tried to construct advanced nanosystems to catalyze intracellular H_2_O_2_ for ROS generation. Here, the H_2_O_2_ is used as chemical stimuli, and such a chemical‐stimuli‐triggered ROS‐generation cancer therapeutic modality with high tumor selectivity was termed as CDT.^[^ ^5^ ^]^ Based on reaction kinetics, H_2_O_2_ decomposition for ROS‐induced toxic efficacy can be divided into the following two methodologies. The first category is spontaneous ROS generation mainly via Fenton or Fenton‐like reaction of nanocatalyst. Common Fenton or Fenton‐like catalysis in ROS‐related therapy includes various nanomaterials with versatile metal ions (e.g., Fe^2+^, Mn^2+^, and Cu^+^), which can decompose intracellular H_2_O_2_ for toxic ^•^OH generation and then lead to oxidative damage of cells.^[^ ^35^, ^189^, ^190^, ^191^, ^192^, ^193^ ^]^ For example, Lin et al. synthesized MnO_2_‐coated mesoporous silica NPs (MS@MnO_2_ NPs) to realize CDT.^[^ ^194^ ^]^ The MnO_2_ shell can react with endogenous GSH, resulting in GSH depletion and Mn^2+^ generation. Then the released Mn^2+^ exhibited strong Fenton‐like performance to catalyze H_2_O_2_ for ^•^OH generation, thereby inducing effective cancer cell killing. In Fenton reaction, the reaction kinetics strongly depends on the reaction parameters such as the H_2_O_2_ level within tumor that may be still not high enough to generate plenty of ^•^OH to damage tumor cells. Therefore, it is greatly significative to improve the level of H_2_O_2_ within tumor to accelerate the Fenton reaction. In this regard, an alternative strategy is to generate H_2_O_2_ in situ by employing glucose oxidase (GOD, enzyme catalyst), since GOD can utilize the abundant glucose within tumor to generate H_2_O_2_ for facilitating ^•^OH generation. A representative work by Huo et al. reported a nanocomposites consisting of natural GOD and ultrasmall Fe_3_O_4_ NPs (inorganic nanozyme, Fenton reaction catalyst).^[^ ^195^ ^]^ The GOD and ultrasmall Fe_3_O_4_ NPs were integrated into the large pore‐sized and biodegradable dendritic silica NPs (GOD‐Fe_3_O_4_@DMSNs nanocatalysts, GFD NCs) (**Figure** **14**a). Here, GOD can effectively deplete glucose within cancer cells to generate a large number of H_2_O_2_ for enhancing the Fe_3_O_4_ NPs‐induced Fenton‐like reaction, and then highly toxic ^•^OH formed via these catalytic reactions results in the apoptosis or necrosis of cancer cells. In recent years, GOD‐mediated Fenton or Fenton‐like reaction have been widely applied in ROS‐induced tumor therapy.^[^ ^189^, ^196^, ^197^, ^198^ ^]^

The second category is the catalytic reaction in response to exogenous stimuli such as light and X‐ray. In Fenton reaction for ROS generation, besides the H_2_O_2_ content, the reaction kinetics is also strongly relied on the rate of redox cycle of Fenton's reagent. For example, in Fe‐based Fenton reaction, the rate of Fe^3+^ reduction is much slower than that of Fe^2+^ oxidation. Therefore, Fe^3+^ is quickly accumulated during the Fenton reaction, which hinders the Fe^3+^/Fe^2+^ cycle and slows the Fenton reaction rate.^[^ ^199^ ^]^ To address the challenge, one of the most common strategies is to employ light to promote the recycling of Fenton reaction.^[^ ^200^, ^201^ ^]^ For example, Ju et al. reported that the incorporation of Cu^2+^ and g‐C_3_N_4_ nanosheets (Cu^2+^‐g‐C_3_N_4_) can result in an intensified photo‐Fenton reaction.^[^ ^200^ ^]^ In the work, Cu^2+^‐g‐C_3_N_4_ can be transformed into Cu^+^‐g‐C_3_N_4_ with visible light irradiation, accelerating ^•^OH generation by Fenton‐like reaction and then supplementing the damage to tumor cells of g‐C_3_N_4_‐mediated PDT. Nevertheless, short‐wavelengths such as visible and UV light, have limited penetration depth in tumor treatments. In this regard, Fenton nanocatalysts with penetration‐enhanced NIR absorbance have exhibited a promising potential for deep‐seated photo‐Fenton therapy. For example, Hu et al. designed UCNP core with mesoporous silica shell to load and deliver Fenton reagent (Fe^2+^).^[^ ^201^ ^]^ Under NIR irradiation, the UCNP cores can convert NIR light to UV or Vis light, and the UV or Vis light can promote the recycling between Fe^3+^ and Fe^2+^. Then the accumulated H_2_O_2_ within intratumoral mitochondrion will be efficiently decomposed into ^•^OH in the presence of Fe^2+^, causing cell damage. In another representative work, Wang et al. reported the biomimetic nanocatalysts consisting of ultra‐small Cu_2−_ *_x_*Se (CS) NPs, GOD and tumor cell membrane (CM), which can realize H_2_O_2_‐supplementary and NIR‐triggered Fenton reaction to kill cancer cells (Figure 14b).^[^ ^189^ ^]^ Here, GOD can increase the content of H_2_O_2_ via in situ catalyzing the glucose in cancer cells. Then, under NIR‐II irradiation, the Fenton reaction‐mediated by CS NPs is drastically enhanced due to the strong NIR localized surface plasmon resonance (LSPR)‐induced electron energy transfer between Cu^2+^ and Cu^+^, which can lead to obvious enhancement in therapeutic effect of breast cancer. In terms of NIR, its penetration ability fails to meet the demand of more deep‐seated therapy. Therefore, developing Fenton nanocatalysts with the deeper‐penetration ability is very significant. In this regard, Zhang et al. reported the Cu_2_(OH)PO_4_ NPs for ROS generation by X‐ray‐triggered Fenton‐like reaction, which can effectively kill tumor cells.^[^ ^202^ ^]^ The enhanced Fenton‐like reaction is attributed to the generation of Cu^+^ active sites on the surface of Cu_2_(OH)PO_4_ NPs because X‐ray can induce electron transfer process in Cu_2_(OH)PO_4_ NPs. In addition to Fenton nanocatalysts, some semiconductor photocatalytic nanomaterials can also catalyze H_2_O_2_ into highly toxic ROS under X‐ray irradiation due to their matched energy band structure. For example, Gu et al. have made many explorations on this aspect. And they have developed many novel semiconductor‐catalytic nanomaterials to apply in triggering H_2_O_2_ for ROS production under X‐ray irradiation, such as BiOI QDs, Au‐Bi_2_S_3_ NPs and bismuth heteropolytungstate [BiP_5_W_30_O_110_]^12‐^ (BiP_5_W_30_) nanoclusters.^[^ ^203^, ^204^, ^205^ ^]^

##### ROS Generation Based on Nanocatalyst with Water‐Splitting Ability

Water splitting to enhance O_2_‐free ROS generation in the presence of a semiconductor catalyst is a novel strategy in ROS‐induced toxic therapy. The basic mechanism of ROS generation by water splitting based on semiconductor catalyst involves the electron transfer from the VB to the CB that are driven via absorbing the electromagnetic radiation, leaving a positive hole in the VB and forming e^−^–h^+^ pairs.^[^ ^206^ ^]^ The fate of e^−^–h^+^ pairs may encounter recombination or migrate to the particle surface. When migrating to the surface of particle, the conduction‐band e^−^ can combine with an electron acceptor with a more positive electrochemical reduction potential than the conduction band edge potential. And the valence‐band h^+^ may react with electron donor with a less positive electrochemical reduction potential than the valence band edge potential.^[^ ^207^ ^]^ The process leads to the reduction of electron acceptor and the oxidation of electron donor. In general, the e^−^ together with h^+^ react with adjacent molecules (O_2_, H_2_O, or OH^−^), resulting in the formation of ROS (^•^O_2_^−^, ^•^OH, or H_2_O_2_).^[^ ^208^ ^]^

**Figure 15:**
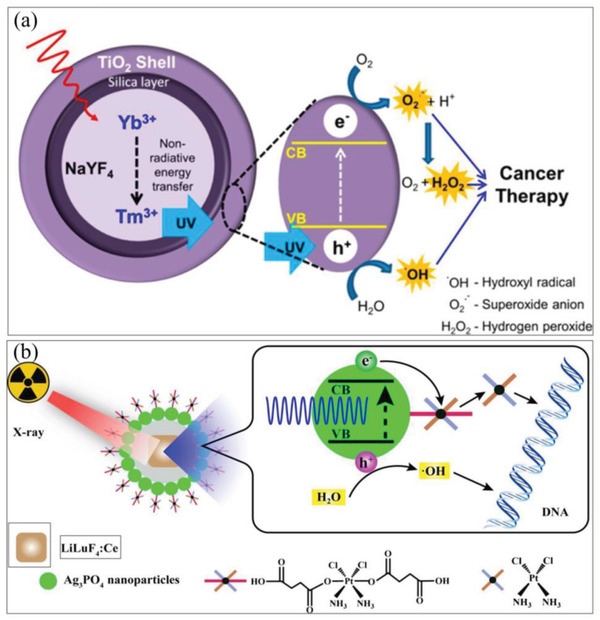
a) Photocatalytic killing schematics of TiO_2_‐UCN. Reproduced with permission.^[^ ^216^ ^]^ Copyright 2015, American Chemical Society. b) Mechanisms underlying the effects of X‐ray‐induced toxic therapy with LiLuF_4_:Ce@SiO_2_@Ag_3_PO_4_@Pt(IV) NPs (LAPNP). Reproduced with permission.^[^ ^223^ ^]^ Copyright 2018, American Chemical Society.

Recent advances in ROS generation based on semiconductor photocatalyst have caused extensive concerns in ROS‐induced toxic therapy. Currently, several types of NPs such as ZnO, TiO_2_, Bi_2_MoO_6_, BiOCl, and SnO_2_ have been developed as effective antibacterial and anticancer agents due to their photocatalytic effects on ROS elevation by water splitting.^[^ ^209^, ^210^, ^211^, ^212^, ^213^ ^]^ For example, Rozhkova et al. indicated that photocatalytic TiO_2_ covalently combining with an antibody via a dihydroxybenzene bivalent linker can trigger phototoxicity to glioblastoma cell. Here, the linker enables UV‐excitated TiO_2_ NPs with absorption of a visible light, thereby realizing visible light‐activated ROS generation.^[^ ^215^ ^]^ Nevertheless, the excited UV/Vis light exhibits a relatively shallow penetration depth for most of semiconductor photocatalyst. Furthermore, UV light itself may have enough energy to cause damage to normal tissues. Therefore, in this regard, the deep penetrating NIR is concerned in the field. In general, narrowing the band gap and red‐shifting the absorption edge is the most common strategy to endow semiconductor photocatalyst with long‐wavelength absorption such as NIR absorption. However, narrow‐band semiconductor photocatalyst under NIR irradiation tends to induce photothermal effect due to rapid recombination of e^−^–h^+^ pairs, leading to low‐effective catalytic performance. Fortunately, in recent years, UCNPs provide a new opportunity for photocatalytic nanomaterials to realize NIR‐activated ROS generation. Currently, the application of NIR‐excitable UCNPs integrating with photocatalytic nanomaterials has demonstrated robust potential in the treatment of cancer because of their ability in penetrating thick tissue.^[^ ^32^, ^213^, ^216^, ^217^, ^218^ ^]^ As a typical paradigm, Lucky et al. coated a photocatalyst‐titanium dioxide (TiO_2_) on a NaYF_4_: Yb, Tm UCNPs core (TiO_2_‐UCNPs) to fabricate a ROS‐generated nanoplatform for PDT (**Figure** **15**a).^[^ ^216^ ^]^ Under 980 nm laser irradiation, upconverted UV light emitted from UCNPs core can excite electrons in the VB of the TiO_2_ shell to the CB, thus inducing the formation of photogenerated e^−^–h^+^ pairs. The reaction of e^−^ and h^+^ with ambient H_2_O and O_2_ molecules promotes a large number of ROS generation, eventually resulting in tumor cell damages.

In addition to NIR light, X‐ray is another important exogenous excitation source for deep‐seated tumor therapy. And X‐ray has been widely applied in organic PSs‐mediated PDT by employing the nanomaterials with unique optical characteristics to attenuate X‐ray into UV‐Vis light such as scintillator nanomaterials (SCNPs).^[^ ^30^, ^219^, ^220^, ^221^ ^]^ A typical work by Chen et al. reported the SCNPs‐mediated nanosystem that is able to realize X‐ray‐induced PDT.^[^ ^30^ ^]^ The nanosystem consists of a SCNPs core and a silica coating loaded with a photosensitizer, merocyanine 540 (MC540). The MC540 can be activated by X‐ray‐induced luminescence of SCNPs for ROS generation and then kill cells. Here, the SCNPs can weaken the high‐energy X‐ray (keV‐MeV) to match the singlet‐triplet energy gap of organic PSs (eV), and also avoid the destruction of PSs by X‐ray. Recently, the strategy is widely used in activating photocatalytic nanomaterials to reduce the dependence of ROS on O_2_ by water splitting for ROS‐induced toxic therapy.^[^ ^222^, ^223^ ^]^ For example, Zhang et al. presented the Ce^III^‐doped LiYF_4_@SiO_2_@ZnO core‐shell structure, where the downconverted UV fluorescence by nanoscintillator under X‐ray irradiation can trigger ZnO NPs to generate e^−^–h^+^ pairs, ultimately resulting in biotoxic ROS formation by water splitting.^[^ ^222^ ^]^ Considering the rapid recombination of e^−^–h^+^ pairs, some approaches such as employing sacrificial agent have been applied in the process of X‐ray‐activated water splitting. For example, Wang et al. recently synthesized LiLuF_4_:Ce NPs as the scintillator that can enable the semiconductors Ag_3_PO_4_ activation to generate electrons and holes under X‐ray irradiation.^[^ ^223^ ^]^ In the work, a cisplatin prodrug (Pt(IV)) was used as an electron sacrificial agent to react with e^−^ and inhibit the recombination of photogenerated e^−^–h^+^, which can increase both the performance of water splitting and the effects of ^•^OH (Figure 15b). Then Pt(IV) is transformed into cisplatin to enhance the level of DNA damage caused by ^•^OH.

**Figure 16:**
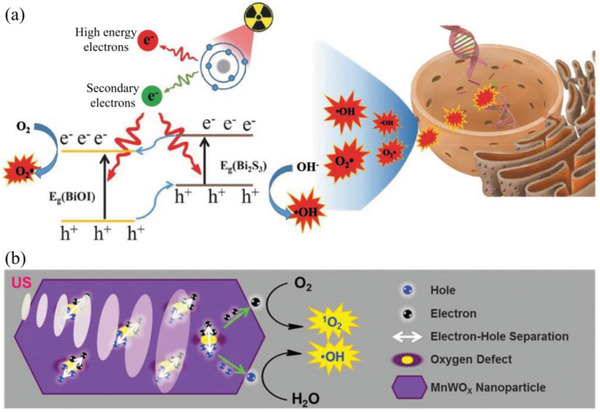
a) The scheme of X‐ray‐induced photodynamic therapy mechanism. Reproduced with permission.^[^ ^225^ ^]^ Copyright 2017, WILEY‐VCH. b) The proposed mechanism of ROS generation by MnWO*_X_* NPs under US irradiation. Reproduced with permission.^[^ ^229^ ^]^ Copyright 2019, WILEY‐VCH.

Furthermore, in recent years, some semiconductor nanomaterials with high‐Z element as radiocatalysis that is able to attenuate the X‐ray energy and directly promote the water splitting for ROS generation also attract extensive attention for efficient cancer therapy. For example, Zang et al. reported that poly(vinylpyrrolidone) (PVP)‐modified Bi_2_WO_6_ nanoplates, which exhibits strong radiocatalytic activity for water splitting and ROS generation under X‐ray irradiation.^[^ ^224^ ^]^ The results indicated that Bi_2_WO_6_ nanoplates can effectively enhance radiotherapeutic efficacy. Similarly, in order to further enhance the catalytic activity of radiocatalysts, improving the utilization levels of X‐ray‐induced e^−^ and h^+^ is an essential strategy. Nanoheterostructures have been developed to address this issue. For example, Guo et al. designed semiconductor heterojunction NPs consisting of two different semiconductors (BiOI and Bi_2_S_3_) to enhance water splitting for ROS generation by promoting the separation of e^−^–h^+^ pairs (**Figure** **16**a).^[^ ^225^ ^]^ In the work, due to the matching energy level structure, the electrons in the CB of Bi_2_S_3_ can migrate to the CB of BiOI and the holes in the VB of BiOI can move to the VB of Bi_2_S_3_. And then the ROS can be effectively generated in the CB of BiOI (reduction reaction) and the VB of Bi_2_S_3_ (oxidation reaction). Such semiconductor heterojunction nanomaterial as radiocatalyst provides a new avenue for the development of deep‐seated and ROS‐mediated cancer therapy based on water splitting. In another work, Cheng et al. designed hybrid nanostructures composed of Au and TiO_2_ NPs to enhance the radiation effect.^[^ ^226^ ^]^ The Au component in the dumbbell‐like Au‐TiO_2_ NPs can generate a lot of secondary photons or electrons under X‐ray irradiation for enhanced radiation effect. Furthermore, those X‐ray‐generated electrons can migrate over the Au‐TiO_2_ NPs interface to the TiO_2_, facilitating the generation of ROS on the TiO_2_ surface.

In addition to current triggering sources such as laser and radiation, US with deep tissue‐penetrating ability and non‐invasiveness has also been employed in cancer therapy to activate semiconductor nanomaterials for toxic ROS generation. Here, semiconductor nanomaterials can be regarded as sonosensitizers to trigger water splitting for SDT. Comparing to traditional organic sonosensitizers such as well‐known hematoporphyrin and Rose Bengal, inorganic semiconductor nanomaterials exhibit high stability and biocompatibility, which are expected to promote the development of SDT. TiO_2_ NPs is one of the most common inorganic semiconductor sonosensitizers, which has been successfully proved to be highly effective in SDT of tumor.^[^ ^227^ ^]^ Similar to light‐ and X‐ray‐triggered ROS generation, fast combination of US‐induced e^−^–h^+^ pair of semiconductor nanomaterials results in the low quantum yield in generating ROS. Therefore, novel inorganic sonosensitizers that is able to inhibit the recombination of e^−^–h^+^ pair is highly ideal. In this regard, a typical example is that Deepagan et al. synthesized hydrophilized Au‐TiO_2_ nanocomposites (HAu‐TiO_2_ NCs) as sonosensitizers to enhance SDT.^[^ ^228^ ^]^ Comparing to pure TiO_2_ NPs, this metal‐semiconductor NCs can improve ROS level by promoting the energy absorption and inhibiting the e^−^–h^+^ pair recombination, and thus effectively suppress the growth of tumor. In another work, Gong et al. reported MnWO*_X_* NPs with oxygen‐deficient structure, exhibiting highly efficient generation of ^1^O_2_ and ^•^OH upon the US trigger (Figure 16b).^[^ ^229^ ^]^ In this work, the oxygen‐deficient structure in MnWO*_X_* NPs can be used as an electron trap site to prevent e^−^–h^+^ pair recombination, effectively promoting ROS generation. Meanwhile, MnWO*_X_* NPs can deplete intracellular GSH, which further enhances the SDT effect. Although inorganic semiconductor nanomaterials as sonosensitizers are still in the initial developing stage, it provides new direction to develop effective sonosensitizers for SDT.

From the above strategies of ROS generation by water splitting based on semiconductor catalyst, inhibiting the rapid recombination of e^−^–h^+^ pair is crucial for ROS generation. Introduction of defects, sacrificial agent employment and metal or semiconductor hybridization in these nanomaterials may be the most promising approaches to obtain considerable catalytic performance for water splitting‐induced ROS generation. In addition, comparing with H_2_O_2_, water splitting for O_2_‐free ROS generation has an advantage in source. Nevertheless, due to the overexpressed H_2_O_2_ in tumor, H_2_O_2_‐participant ROS generation is more effective in tumor cells but not in the normal tissue, which can selectively kill cancer cells. While H_2_O‐participant ROS generation can happen in both normal and tumor cells, exhibiting nonspecific killing during treatments. Therefore, in terms of nanomaterials for water splitting to generate ROS, it needs to enhance their tumor targeting to avoid adverse effect on normal cells.

## 3 Principles for the Design of ROS‐Associated Nanomedicines

ROS‐regulating nanomedicines play a vital role in ROS‐related disease treatments. ROS‐downregulating nanomedicines can be used as ROS scavenger for antioxidant therapy. And ROS‐upregulating nanomedicines can induce oxidative stress injuries for antineoplastic therapy. The study of ROS‐associated nanomaterials has been around for many years and a large amount of information was obtained during the progress. However, most of the studies mainly focus on getting the nanomedicines to work, but not on the development and summary of design aspects in ROS‐regulating nanomedicines. A major challenge in the promotion of design strategies is that both nanomaterials with potential in the incorporation of ROS‐related nanomedicines and their activation manners are extremely diverse. Therefore, it is necessary to establish useful general guidelines on how to minimize the effort for the further optimization of each case during the design of ROS‐regulating nanomedicines. In this section, according to the strategies in ROS elimination and elevation discussed above, we simply propose several prioritized and important principles that may have the potential to provide the initial framework for technicians and scientists to design and optimize the ROS‐regulating nanomedicines. And these principles to ultimately improve the therapeutic effect of ROS‐associated nanomedicines can be roughly divided into three categories: 1) Principles specific for ROS regulation; 2) Principles in nanostructure optimization; 3) Principles for the responses to biological challenges.

### 3.1 Principles Specific for ROS Regulation

#### 3.1.1 Boosting O_2_ Elevation

In ROS‐enhaced disease treatments, especially in tumor therapy, inadequate O_2_ supply severely limited ROS generation of some therapeutic modalities such as PDT, RT and SDT. Currently, some strategies mainly involving exogenous oxygen delivery and oxygen self‐supplement based on nanomaterials are used to elevate O_2_ of hypoxic tumor. For O_2_‐delivery nanosystems, their development are hindered by only a narrow range of O_2_ carriers. Common O_2_ carriers mainly include PFC, Hb and MOFs.^[^ ^149^, ^150^, ^160^ ^]^ And most of them may face with premature release of O_2_, transient generation of O_2_ or limited O_2_ capacity. In recent years, some efforts have been devoted to optimize O_2_‐delivery nanosystems. For example, reoxygenation of O_2_ carrier is developed to improve the O_2_ capacity and ensure the continuous O_2_ delivery.^[^ ^153^ ^]^ And exogenous stimuli is introduced into O_2_‐delivery nanosystems to realize controllable oxygen release.^[^ ^154^ ^]^ For oxygen self‐supplement, it can accomplish O_2_ generation in situ with the assistance of nanomaterials. In these strategies, these O_2_‐evolving nanomaterials that can catalyze endogenous H_2_O_2_ and H_2_O for O_2_ generation are particularly noteworthy. Because it provides a effective strategy to locally generate O_2_, which can avoid premature leakage of O_2_ comparing to O_2_‐delivery nanosystems. Nevertheless, in H_2_O_2_‐triggered O_2_ elevation, the intrinsically limited H_2_O_2_ level in tumor may dramatically restricts the O_2_ generation. Therefore, regulating the H_2_O_2_ level of TME may be one of the most effective strategies to guarantee the H_2_O_2_‐mediated O_2_ elevation. While in H_2_O‐triggered O_2_ elevation, the most important thing is to develop high‐efficient catalysts that can decompose H_2_O into O_2_. Seen from recent studies in O_2_ elevation, it can be found that most of the studies mainly focus on getting the new nanosystems to generate O_2_, but not on the spatial relationship between the distribution of O_2_ molecule and hypoxic tumor region. Since the penetrability of O_2_‐elevating nanomaterials and O_2_ inside tumor are limited, it is very important to optimize the spatial distribution of nanomedicines. In addition, in ROS‐enhanced nanosystems based on O_2_ elevation, the appropriate time after tumor reoxygenation to trigger ROS generation needs to be explored because the high‐level O_2_ can maximize the ROS generation.

#### 3.1.2 Enhancing the Generation and Elimination of Specific ROS

In ROS‐enhanced toxic therapy, common ROS mainly include ^•^O_2_^−^, H_2_O_2_, ^1^O_2_, ONOO^−^, ^•^OH, etc. Among those ROS, ^•^O_2_^−^ is a poor oxidant and has a low reactivity to most biomolecules. It is mainly generated by the conversion of O_2_ molecules. In general, the toxic effects of ^•^O_2_^−^ are mainly due to the conversion of ^•^O_2_^−^ to a more reactive radical such as ^•^OH and ONOO^−^.^[^ ^20^, ^230^ ^]^ Similarly, H_2_O_2_ is also an oxidant with poor reactivity, but unlike the ^•^O_2_^−^, H_2_O_2_ can easily cross cell membranes to serve as a critical signaling molecule.^[^ ^5^ ^]^ And the biologically damaging effects of H_2_O_2_ are attributed to secondary products such as ^•^OH.^[^ ^2^ ^]^ For ^1^O_2_, it can directly oxidize proteins, DNA, and lipids. However, the generation of ^1^O_2_ is highly dependant on the level of ambient O_2_, just like that of ^•^O_2_^−^. Besides that, another considerable ROS is ONOO^−^ that can be generated via the reaction of nitric oxide radical and ^•^O_2_^−^, because ONOO^−^ is a powerful oxidizing and nitrating agent.^[^ ^230^ ^]^ For ^•^OH, it is one of the most reactive radicals and has a very short half‐life in vivo, exhibiting rapid and indiscriminate damage to biomolecules.^[^ ^2^ ^]^ Therefore, from the perspective to obtain O_2_‐free and highly reactive ROS, specifically to promote ^•^OH generation with the assistance of nanomaterials may be more conducive to improve the therapeutic efficacy of toxic therapy.

In nanomaterials‐mediated antioxidant therapy, most of these nanomaterials exhibit strong scavenging ability for different ROS. The broad ROS‐scavenging ability of a nanomaterial can endow it with promising potential in various diseases induced by different ROS. For example, a nanomaterial with ^•^OH‐ and ^•^O_2_^−^‐scavenging ability may be applied in radiation injury (^•^OH is believed to be one of the major triggers of radiation injury) and rheumatoid arthritis (SOD‐like drug can reduce the damage in rheumatoid arthritis by quenching free radicals), respectively.^[^ ^231^, ^232^ ^]^ However, besides targeted ROS that needs to be eliminated, these nanomaterials with broad ROS‐scavenging ability may consume other ROS inside cells and then cause tissue dysfunction during the process of antioxidant therapy. Therefore, one of the most promising strategies is to develop nanomaterials with single ROS‐scavenging ability for selective and specific antioxidant therapy. In addition, the nanomaterials for specific ROS elimination can be used to investigate the pathogenesis and figure out the role of ROS in ROS‐related diseases.

#### 3.1.3 Optimizing the Catalytic Performance

Catalytic nanomaterials occupy an important position in ROS‐scavenging and ROS‐enhanced nanomedicines. Therefore, optimizing the catalytic performance is an important subject to accelerate their clinical application. In above section, some key strategies to improve the catalytic performance of ROS‐enhanced nanomedicines that mainly involve some catalytic nanomaterials have been elucidated. Here, aiming at antioxidant nanomedicines, we proposed several opinions that may be beneficial to improve the catalytic property of antioxidant nanomaterials. In general, the catalytic property of a catalyst is dependent on its structural and chemical factors. For example, in many cases, the catalytic activity of a catalyst highly depends on its size and shape. From the surface science point of view, it is because a solid‐liquid or solid‐gas catalytic event is performed on the surface sites of a catalyst by the necessary surface adsorption step.^[^ ^233^ ^]^ While small‐sized NPs with large surface area to volume ratio can provide abundant active sites.^[^ ^234^ ^]^ In addition, when the size of NPs decreases to a certain point, its electronic state and adsorption energy, as well as coordination environment of surface atoms can undergo dramatic changes. For shape effect, the exposed facets associated with the shape of NPs significantly influence their catalytic activity.^[^ ^233^ ^]^ For example, the (111) facets predominant tetrahedral Pt NPs possess high activity on the electron‐transfer reaction between hexacyanoferrate ions and thiosulfate ions, while the (100) facets predominant cubic NPs are least active, which attributes to the larger number of active surface atoms on edges and corners of tetrahedral Pt NPs.^[^ ^233^, ^235^ ^]^ Therefore, the evaluation of the shape and size effect on the catalytic property of ROS‐scavenging nanomedicines is critical to obtain optimal antioxidants.

As mentioned above, nanomaterials with specific chemical structures such as oxygen vacancies and shifting/mixed valence states may be the feasible candidates for ROS elimination. Here, the catalytic activity of these antioxidants is implicated in the concentration of oxygen vacancies or the proportion of different valence states.^[^ ^236^, ^237^ ^]^ For example, a higher number of oxygen vacancies (Ce^3+^/Ce^4+^) in ceria are required in the elimination of ^•^O_2_^−^ and ^•^OH.^[^ ^238^ ^]^ Therefore, the effect of the proportion of different valence states on antioxidant performance should be evaluated. In addition, in order to maintain the catalytic performance of ceria, the reversible Ce^3+^/Ce^4+^ redox cycles need to be guaranteed. In another word, the shifting between Ce^3+^ and Ce^4+^ is important for the antioxidant performance of ceria. In general, electron transport across NPs with mixed valence states under external stimuli such as irradiation can realize the shifting of valence states.^[^ ^239^ ^]^ Similar to the viewpoint in introducing stimuli responsiveness, for some high‐energy external stimuli such as X‐ray and UV that can induce oxidative damage, they are not appropriate for the increasement of catalytic performance of antioxidant nanomedicines. In this section, we proposed that regulating the state of oxygen vacancies or shifting/mixed valence states in NPs may provide a promising strategy to improve the antioxidant performance of catalytic nanomaterials.

#### 3.1.4 Reducing Protein Adsorption

Due to the nanosize and high surface‐to‐mass ratio, recently, it is widely accepted that NPs do not directly interact with living cells but a protein corona (PC) forms on the surface of NPs, which can alter the biological effects of NPs when NPs are introduced to biological milieu. The PC influences the biological progress of NPs, involving the cellular uptake, biodistribution, clearance, toxicity, and immune response.^[^ ^240^, ^241^ ^]^ For example, Cheng et al. studied the roles of PC on cellular internalization of different‐sized gold NPs. The results indicated that PC can obviously reduce the uptake of Au NPs with a cell type‐ and particle size‐dependent manner. And the PC can inhibit the internalization of large‐sized Au NPs compared with small‐sized Au NPs.^[^ ^242^ ^]^ Besides that, the PC composed of proteins and other biomolecules can reduce the surface energy of NPs by adsorbing on their surface.^[^ ^243^ ^]^ In other words, the adsorbed proteins can shield the NPs surfaces and prevent the diffusion of ROS to cellular components, and thus may reduce the oxidative stress damages to cells.^[^ ^243^ ^]^ Therefore, from the view of enhancing the surface activity of ROS‐associated nanomedicines, it is feasible and essential to mask nanomedicines and prevent them from interactions with endogenous biomolecules for PC formation. In recent years, many methods are used to avoid the protein corona phenomenon. Commonly adopted strategies aiming to reduce protein adsorption include preformed protein coronas and surface grafted non‐charged hydrophilic polymers.^[^ ^244^ ^]^ However, for preformed protein coronas, it seems to be not appropriate in the application of ROS‐related disease treatments due to its additional shielding effect for NPs. While for non‐charged hydrophilic polymers, they can reduce interactions with plasma components but not influence the surface activity, which attributes to the decrease of the hydrophobic interactions and electrostatic forces between the proteins and the surface in solutions.^[^ ^244^, ^245^ ^]^ Currently, some polymers such as the poly(methyl methacrylate) (PMMA) and PEG are often used to inhibit the protein adsorption because of their near‐neutral and hydrophilic properties.^[^ ^245^ ^]^

#### 3.1.5 Regulating Cellular Reaction Parameters

From current results of nanomaterials‐mediated antioxidant therapy, the antioxidant activity of the majority of nanomedicines depends on its nature such as chemical structure and catalytic activity, and has less dependence upon the cellular reaction environment except for endogenous‐stimuli responsive antioxidant delivery nanosystem. While for most of ROS‐enhanced nanomedicines, they are heavily dependent on the cellular reaction environment such as pH value, O_2_ and H_2_O_2_ concentration, as well as the level of cellular antioxidant such as GSH.^[^ ^194^, ^195^, ^202^, ^246^ ^]^ In above section, we have discussed related strategies to improve cellular O_2_ and H_2_O_2_ level and then enhance the ROS generation. Besides O_2_ and H_2_O_2_, pH range also has a huge impact on the ROS generation. Many studies indicated that the ROS‐generated capacity of catalytic nanomaterials is related to the pH value.^[^ ^247^, ^248^ ^]^ In previous report, researchers successfully regulated intracellular pH by MCT4 silencing. The MCT4 silencing can inhibit the efflux of lactate/H^+^ generated by glycolysis of tumor to induce acidosis of tumor cell, meanwhile, result in oxidative damages to tumor cells by enhancing the Fenton‐like reaction.^[^ ^249^ ^]^ It can be seen that regulating pH value to optimize reaction parameters of catalytic nanomedicines may be a potential approach to enhance ROS‐induced toxic therapy. Nevertheless, in biological system, it is difficult to regulate the intracellular pH level. Thus, pH‐regulating strategies have not been extensively applied in ROS‐related therapy modalities. In fact, pH regulation is not the only strategy to obtain an ideal pH environment for ROS enhancement. It is well known that the pH value of the TME is around 6.5–7, the endosomes and lysosomes in tumor cells exhibit a pH value in approximately 5.0 and 4.5, respectively.^[^ ^35^ ^]^ Therefore, developing a strategy to precisely deliver the nanomedicines to these sites in tumor with appropriate pH value may also enhance ROS generation.

In addition to pH range, another key element is the endogenous antioxidant system such as SOD and GSH.^[^ ^250^, ^251^ ^]^ These antioxidant molecules can counteract the increase of ROS induced by nanomaterials, which may result in the total ROS levels below the toxic threshold and influence the therapeutic effect. Therefore, it is an ideal manner to attenuate the antioxidant system before ROS generation. Typically, Ma et al. synthesized in situ GSH‐activated self‐assembled copper‐amino acid mercaptide NPs (Cu‐Cys NPs) for ROS‐toxic therapy.^[^ ^252^ ^]^ In detail, after entering into tumor cells, the Cu‐Cys NPs first reacted with intracellular GSH, inducing GSH depletion and reducing Cu^2+^ to Cu^+^. Subsequently, the formed Cu^+^ can trigger the Fenton‐like reaction to catalyze intracellular H_2_O_2_ and produce highly toxic ^•^OH. In the work, GSH depletion and ^•^OH generation cause lipid peroxidation, protein inactivation, DNA damage and ultimate cell apoptosis, providing a promising strategy to break antioxidant defense system of cell for ROS elevation. In terms of ROS‐generation nanomedicines, regulating cellular reaction parameters is just in the beginning stages, and further substantial efforts in this field will greatly facilitate the development of more robust nanomedicines for ROS‐toxic therapy.

#### 3.1.6 Employing High‐Z Element‐Based Nanomaterials

Nanomaterials containing high‐Z elements under X‐ray irradiation exhibit strong photoelectric absorbance capacities, which can release electrons (photoelectrons and Auger electrons) for subsequent ROS generation. These high‐Z element‐based nanomaterials, such as gold NPs, rare earth NPs, Bi‐based NPs, W‐based NP, Hf‐contained NPs, etc., have shown the ability to generate a lot of ROS for enhanced radiation effect.^[^ ^5^, ^253^ ^]^ In addition, these electrons released from high‐Z nanomaterials with X‐ray irradiation can not only directly promote ROS generation and interact with near tumor tissues, but also activate adjacent semiconductor nanomaterial to enhance the migration of electrons and holes for further ROS improvment. For example, in above‐mentioned work, the Au component in the dumbbell‐like Au‐TiO_2_ NPs can generate a lot of secondary photons or electrons under X‐ray irradiation to facilitate the generation of ROS on the TiO_2_ surface.^[^ ^226^ ^]^ Besides that, in recent years, many studies indicated that semiconductor nanomaterial with high‐Z elements can directly be activated by X‐ray for ROS generation via a radiocatalytic process, such as BiOI QDs, Bi_2_WO_6_, WO_3_‐Ag_2_WO_4_ NPs, Au‐Bi_2_S_3_ NPs and bismuth heteropolytungstate [BiP_5_W_30_O_110_]^12‐^ (BiP_5_W_30_) nanoclusters, etc.^[^ ^203^, ^204^, ^205^, ^254^ ^]^ In the radiocatalytic process, these semiconductor nanomaterial can generate a lot of e^−^–h^+^ pairs under X‐ray irradiation. These highly active e^−^–h^+^ pairs can react with adjacent molecules such as O_2_ and H_2_O to enhance ROS generation. In ROS‐enhanced therapy, employing high‐Z element‐based nanomaterials with X‐ray irradiation provides a simple and effective strategy for ROS enhancement. Currently, high‐Z nanomaterials for RT exhibit huge potential for tumor therapy. And hafnium oxide NPs (NBTXR3) as the first nanomedicine for ROS‐related therapy have been approved in market.

### 3.2 Principles in Nanostructure Optimization

#### 3.2.1 Preventing the Leakage of Scavengers

During transit of nanocarriers for delivery of ROS scavenger or PSs, it is possible that these drugs are prematurely released from the nanomaterials before reaching the targeting sites, which may potentially reduce the therapeutic activity or cause adverse effects. In recent years, some strategies are developed to stabilize the nanomaterials‐based drug delivery platform, which can be a reference to the delivery of ROS scavengers or PSs to minimize their premature release. Enhancing the interaction such as hydrophobic interaction, electrostatic interaction, van der Waals force, *π*–*π* stacking, hydrogen and covalent bonding, as well as nano‐encapsulation between drug and nanomaterials is one of the most common strategies.^[^ ^166^, ^255^, ^256^ ^]^ Here, the loading strategies based on hydrogen bonding, van der Waals force and electrostatic interaction have been widely applied in drug delivery. However, they may still be exposed to leakage risk because the drugs are weakly bound to these NPs. In recent years, the covalent bonding and nanoencapsulation are believed to be the most reliable way to avoid leakage of scavengers or PSs. Covalent conjugation strategies allow the drug release by cleaving conjugated bonds to prevent premature release in the absence of a precise trigger.^[^ ^257^, ^258^, ^259^ ^]^ However, the covalent conjugation is a complicated synthetic process because it may need the design of prodrug with appropriate chemical group to covalently bound to the nanomaterials. For nano‐encapsulation, it mainly involves the hollow nanomaterials to encapsulate drug, and then the mesopore or cavity is capped with a biodegradable shell or particle.^[^ ^260^, ^261^, ^262^, ^263^, ^264^ ^]^ For example, MnO_2_ and ZnO are usually used as gatekeepers to effectively reduce the leakage of drug, which attributes to that they can be removed at the acid intercellular environment.^[^ ^261^, ^264^ ^]^ The above relatively mature strategies can provide a decent frame of reference to construct the delivery nanoplatform of antioxidants or PSs.

#### 3.2.2 Introducing Stimuli Responsiveness

In recent years, smart nanomedicines are proposed to precisely realize maximum theranostics by responding to specific stimuli such as light, redox potential, and pH. Stimuli‐responsive systems are first suggested in drug delivery system. Currently, some non‐drug delivery system that completes active treatments only upon exposure stimuli such as NIR, external X‐ray and magnetic field can also be classified as stimuli‐responsive system.^[^ ^265^ ^]^ In fact, in terms of antioxidants or PSs delivery nanosystem, introducing specific stimuli can also reduce the leakage of loaded antioxidants or PSs. However, compared with some of above‐mentioned strategies such as covalent bonding and nanoencapsulation, the biggest advantage of stimuli‐responsive delivery systems is spatiotemporally controlled sites targeting to realize local drugs release, which may provide more reliable and efficient approaches to trigger drug release. In this regard, the design of nanocarriers that can respond to endogenous or exogenous stimuli may provide a promising alternative to realize targeted delivery of drugs. In general, common endogenous stimuli that can be applied in the design of ROS‐regulating nanomedicines include the concentrations of enzymes, redox potential or pH.^[^ ^265^, ^266^ ^]^ Here, the low pH is a common feature in many ROS‐related diseases such as inflammatory and cancer environments.^[^ ^267^, ^268^ ^]^ And the redox regulation is the ultimate goal for ROS‐related disease treatments. While the altered expression profile of specific enzymes is varied in different pathological conditions. Therefore, taken together, pH and redox‐sensitive nanosystems are more likely to realize a broad spectrum of ROS‐related therapy. Currently, pH‐sensitive nanoplatforms have been widely applied in PSs delivery, which can release the loaded PSs or recover the toxicity of PSs in weak acid.^[^ ^269^, ^270^ ^]^ And the pH‐responsive delivery in antioxidant began to gradually rising, causing widespread concern.^[^ ^271^, ^272^ ^]^ For example, pH‐sensitive polymeric NPs based on poly(VI‐co‐HEI) with dexamethasone have been used to prevent the cisplatin‐induced hearing loss.^[^ ^271^ ^]^ For a redox‐sensitive nanoplatform, it exhibits considerable potential for antioxidant application. Because the ROS depletion happens in the whole release process of antioxidants, and then the released antioxidants conduct the supplement therapy by effectively eliminating excess ROS. In addition to endogenous stimuli, exogenous stimuli involving X‐ray, heat, magnetic field, and US can also be used to trigger drug release.^[^ ^265^ ^]^ However, in the delivery of antioxidants, it needs to consider the effect of exogenous stimuli on ROS generation. Because some high‐energy light such as UV and X‐ray may induce additional oxidative damage.^[^ ^273^, ^274^ ^]^ When the exogenous stimuli are introduced in the antioxidant delivery system, it is believed that magnetic field and US with deep‐tissue penetration should be identified as priorities because they may not lead to additional oxidative damage. Furthermore, for non‐drug delivery system such as ROS‐upregulating nanosystem, current results indicated that exogenous stimuli such as X‐ray and US are attractive in designing advanced stimuli‐driven nanomedicines for deep‐seated ROS‐toxic therapy.

#### 3.2.3 Endowing the Active‐Targeting Ability

Non‐specific distribution of nanomedicines is one of the biggest obstacles for their clinical application, which will result in the loss of therapeutic effect and severe side effects to normal tissue. For active‐targeting nanomedicines, they can complete site‐specific delivery via modifying targeting ligands on their surface, in which the targeting ligands can recognize receptors overexpressed in pathological tissues and then facilitate the selective uptake of nanomedicines.^[^ ^275^, ^276^ ^]^ And many targeting ligands such as aptamers, nanobodies, small molecules and peptides have been used in active‐targeting nanosystems.^[^ ^277^, ^278^, ^279^, ^280^ ^]^ Currently, tumor‐targeted nanomedicines is one of the most widely studied topics because tumor tissues can provide abundant signals as targets to construct various nanomedicines with active‐targeting ability. The human epidermal growth factor receptor‐2 (HER2), transferrin receptor (TfR), prostate‐specific membrane antigen (PSMA) and epidermal growth factor receptor (EGFR) as well‐established targets have been applied in the clinical‐trial stage of cancer therapy.^[^ ^277^, ^281^, ^282^ ^]^ Since most of ROS‐elevation nanomedicines are used to treat tumor, many existing strategies in targeting tumor can provide a reference for the design of ROS‐enhanced nanomedicines with targeted ability. In recent years, in order to enhance ROS‐induced toxic therapy, many NPs with active‐targeted ability are developed for improved accumulation in pathological sites.^[^ ^283^, ^284^ ^]^ While ROS‐elimination nanomedicines are mainly applied in non‐tumor diseases such as inflammatory and neurogenic disease, thus these tumor‐targeted strategies may not apply to the targeted modification of antioxidant nanomedicines. Current research in targeted antioxidant nanomedicines is insufficient, and fails to provide valuable information with statistical significance. Therefore, in order to improve the advantage of antioxidant nanomaterials and their ultimate potential for clinical translation, more detailed and extensive studies in this area are necessary.

### 3.3 Principles for the Responses to Biological Challenges

#### 3.3.1 Overcoming the Biological Barriers

Biological barriers can prevent the effective accumulation of nanomedicines to disease sites, inhibiting the exertion of therapeutic effect. In common biological barriers, the nonspecific uptake of nanotherapeutics in healthy organs may be the major limitation of nanodrugs delivery.^[^ ^285^ ^]^ Currently, many strategies have been proposed to overcome the nonspecific uptake by prolonging the circulatory half‐life of nanomedicines.^[^ ^286^ ^]^ For example, the strategy of PEGylation NPs that can hinder the clearance by the reticuloendothelial system (RES) or the mononuclear phagocyte system (MPS) have been applied in clinic.^[^ ^287^, ^288^ ^]^ In addition, some other biological barriers such as dense intercellular matrix (ECM), cellular membrane traversal and BBB are also the innate obstacles to obtain more effective drug delivery. In this regard, many efforts have been devoted to address these barriers, such as normalization of the tumor vasculature and site‐specifically switching the charge of NPs to heighten tumor cell entry.^[^ ^285^, ^289^ ^]^ And most of these approaches can be used as references for the delivery of ROS‐associated nanomedicines. For instance, the BBB, as a major obstacle in various neurological disorders, significantly impedes the delivery of drugs. Currently, various strategies such as chemical modification of drug and prodrugs, NPs‐mediated local delivery, and disruption of the BBB have been developed to address the BBB.^[^ ^290^ ^]^ Here, common NPs that can cross the BBB mainly involve polymeric NPs such as poly(lactic acid) (PLA) NPs, PBCA, liposomes, and PLGA, as well as inorganic materials such as zinc oxide, silver, gold NPs, and quantum dots (QDs).^[^ ^289^ ^]^ Several of them such as PBCA and PLGA have been successfully applied in the delivery of antioxidant across the BBB. ^[^ ^51^, ^52^ ^]^ About biological barriers, they are not adequately addressed at the time of NPs design due to their complexity. And very few NPs can simply face one or a few biological barriers progress. Therefore, sustained attention in overcoming the biological barriers is important and necessary.

#### 3.3.2 Promoting Homogeneous Distribution

The distribution of ROS‐regulating nanomedicines in disease region has a strong impact on the therapeutic effect. Especially in ROS‐induced tumor therapy, the generated ROS have relatively short diffusion distances, and thus it is believed that to seek the homogeneous distribution of ROS‐upregulating nanomedicines in solid tumor can effectively generate ROS in an entire tumor and reduce the survival of tumor cells. However, the abnormal vasculature and dense interstitial matrix in solid tumor result in heterogeneous distribution and poor penetration of drug throughout the entire tumor.^[^ ^291^ ^]^ Some common strategies such as the regulation of TME (e.g., vascular normalization and vascular disruption) and modulation of ECM, as well as optimization of physical properties of NPs such as size, surface properties and shape can be used to enhance the penetration of nanosystems.^[^ ^292^ ^]^ Here, vascular disruption can improve the blood vessel permeability by employing physical forces such as heat, US and radiation, as well as introducing vascular disrupting agents.^[^ ^203^, ^293^, ^294^, ^295^ ^]^ Vascular normalization for restoring blood supply is usually realized by using anti‐angiogenic agents to inhibit rapid formation of dysfunctional vascular networks, ultimately inducing homogeneous distribution of therapeutic agents inside tumor.^[^ ^292^ ^]^ While for the modulation of ECM, it is mainly achieved through blocking the ECM synthesis or depleting ECM with the aid of nanosystems.^[^ ^292^, ^296^ ^]^ Among these strategies, optimization of physical properties of NPs may be relatively easy to control in a demanding manner. In recent years, many works investigated the ideal properties of NPs to enhance their penetration in tumor. In particular, the size of NPs is one of the most important factors in determining the penetration. Therefore, regulating size is widely applied in improving heterogeneous distribution of NPs. In general, smaller size can significantly decrease their diffusional hindrance in the interstitial matrix and allow penetration into the tumor. Meanwhile, they can be rapidly removed by renal excretion, reducing the harm due to long‐term body retention.^[^ ^203^ ^]^ This strategy could apply to intratumor injection but not to intravenous injection, because these ultrasmall NPs with efficient renal clearance fail to accumulate in tumor through the enhanced permeability and retention (EPR) effect by intravenous injection. Recently, size‐tunable NPs are used to overcome the challenge in intravenous injection. The original NPs with large size are capable of being well retained in the tumor surroundings by EPR effect. Then the large‐size NPs are converted to small‐size NPs in response to TME (e.g., enzyme, pH), ultimately penetrating deeply in tumor.^[^ ^297^ ^]^ Despite great efforts in improving the homogeneous distribution of nanomaterials have been made, the more detailed mechanism of penetration is still required because of the complexity of biological barriers. The above strategies can provide an option for the design of ROS‐upregulating nanomedicines with strong tumor penetration ability.

## 4 Conclusions and Outlook

Numerous efforts have been devoted to consolidating the development of ROS‐downregulation and ROS‐upregulation nanomedicines, and a large number of strategies have been developed to address the existing issues in redox‐regulating therapy. In the review, we summarized the current progress of ROS‐associated nanomedicines in disease therapy involving antioxidant therapy and ROS‐induced toxic therapy, presenting several fundamental and key principles for the design of ROS‐associated nanomedicines. Although the development of ROS‐regulating therapy has made significant progress in recent years, the attention in the design of ROS‐regulating nanomedicines is still in the infancy and there remain many challenges to be addressed.

First, due to the increasing attention in the functional modification of ROS‐regulating nanomedicines to seek precise therapy in recent years, complex structural and functional design such as ligand modification and nanocomposite structure will lead to the complexities and difficulties in large‐scale preparation with high repeatability, because slight variations in the manufacturing process may result in dramatic changes in the physical and chemical properties such as surface charge, size, component and crystallinity, even the therapeutic outcomes. From the perspective of large‐scale preparation, we advocate developing simple but effective nanomedicines. Meanwhile, we have also nothing against the multifunctional nanomedicines with complex structure. However, more effort is needed to close the gap between the study in the guiding principles of large‐scale preparation and optimization.

Second, in ROS‐mediated biological events, besides the ROS‐related disease treatments, ROS‐related toxicity induced by nanomedicines should be revealed and regulated. The mechanisms of toxicity for nanomaterials are complex. Nevertheless, an important mechanism of nanotoxicity is the ROS generation by these nanomaterials that can induce the subsequent oxidative stress in tissues. This is primarily because small‐sized nanomaterials have high specific surface area and high surface reactivity compared with their bulk‐size counterparts, thereby resulting in higher levels of ROS generation. Therefore, in order to facilitate the development and application of nanomedicines, it is significative to construct the strategies to reduce the ROS‐related toxicity for safety. However, the mechanisms of nanomaterials‐mediated ROS production are diverse. It is mainly because that the ROS generation by nanomaterials is dependent on their physical and chemical properties such as size, shape, surface area, surface regulation, and degree of aggregation and agglomeration. In addition, the interaction with environmental characteristics such as light and encountered physiological environment are also important factors in ROS‐induced toxicity. Therefore, understanding the possible bio‐physicochemical factors and their influencing rule in ROS generation could provide guidance and critical information on the safe design of nanomedicines. Furthermore, systematic and comprehensive summarization in the currently known strategies that may shed the ROS‐induced toxicity are also important and necessary for the development of safe nanomedicines.

Thirdly, detection techniques of ROS in biological systems should be paid attention to continuously. The generation or elimination of ROS is spatially and temporally dynamic within the cell after introducing nanomaterials, making the study of ROS dynamics and biology particularly difficult.^[^ ^298^ ^]^ In order to measure these dynamics, precise tools and techniques of ROS in biological systems that can specifically detect the relevant ROS are vitally important for the development of ROS‐related nanomedicines. Conventional techniques including electron paramagnetic resonance spectroscopy, mass spectrometry and high performance liquid chromatography are extensively used as analytical tools to detect ROS.^[^ ^299^ ^]^ However, most of these methods can not provide real‐time monitoring, failing to obtain dynamic information and precise quantification about the generation or elimination of ROS. It is necessary to develop relevant detection techniques to address above issues. In recent years, the methods by employing colorimetric, fluorescent and luminescent probes are widely applied in the kinetic evaluation of ROS reactions.^[^ ^5^ ^]^ However, they may be nonspecific and be intervened by other types of ROS during detection. The specific detection of ROS both in vitro and in vivo, especially in vivo, is still a major challenge. Therefore, in the future development of ROS detection, the techniques with the ability of real‐time monitoring and recognition to specific ROS are important and necessary.

Another important effort is the regulation and standard in the performance evaluation of ROS‐regulating nanomedicines. Currently, a large amount of ROS‐related therapy based on nanomedicines have been reported. Although all of the known ROS‐related nanomedicines exhibit considerable therapeutic effect, there is no efficient and uniform method for comparison among those nanomedicines to get the valuable information in efficacy and performance. It is believed that, by using the same standard and evaluation system, the advantages and disadvantages of different nanomedicines can be uncovered, which can provide a judgement in the application potential aiming at different nanomedicines. Thereby, these ROS‐regulating nanomedicines with better performance can be selected and assessed for the future application. Such an evaluation system may save the drug screening time in vast amounts of ROS‐related nanomedicines. In addition, from the comparison among the same kind of nanomaterials, the influence factor on their performance can be obtained, revealing the defects in current design and then offering the next design direction for optimization. We believe the regulation and standard will make a greater contribution to the benign development of ROS‐related nanomedicines during the design stage.

Furthermore, in ROS‐regulating therapy, further improving the reliability of endogenous stimuli‐responsive strategies is crucially important for the development of stimuli‐responsive therapeutic modalities of ROS‐associated nanomedicines. In recent years, employing stimuli‐responsive nanomaterials is a very important strategy to realize maximum theranostics in diseased tissue and reduce the damages of normal tissue. Many publications have reported that stimuli‐responsive nanosystems exhibit distinct advantages in precise drug delivery. It provides a novel and efficient approaches to trigger drug release within diseased tissue. For ROS‐regulating therapy, introducing stimuli‐responsive strategies also improve the advancement of ROS‐associated nanomedicines. Nevertheless, in the development process of endogenous‐responsive nanomedicines, an issue that some diseased tissue may not exhibit an obviously difference in stimuli level comparing with that in normal tissue should be paid attention, as they may receive compromised therapeutic effects because endogenous‐responsive systems fail to accomplish precise and efficient drug release in these diseased tissues. For example, in terms of ROS (redox)‐responsive nanoplatforms, since ROS (redox) level may be varied from living body, their therapeutic effects strongly depend on pathophysiology or type of diseased tissue. Similarly, for other endogenous stimuli‐responsive nanomaterials such as pH‐, enzymes‐ or GSH‐responsive nanomaterials, they may also suffer the same issues. Therefore, further boosting the difference in stimuli level between diseased tissue and normal tissue and ensuring the stimuli level among different pathophysiologies should be highly regarded in the future development of ROS‐regulating therapy. Not only ROS‐regulating therapy, this concern can also be applied to all endogenous stimuli‐responsive therapeutic modalities.

After addressing these challenges in the design of ROS‐regulating nanomedicines, the final concern for the development of this booming field is the clinical translation of these well‐designed nanostructures. Although some nanomedicines have been approved by FDA or NMPA, most of them are organic nanostructures that serve as nanocarriers to load active pharmaceutical ingredients for chemotherapy, gene therapy, or immunotherapy. They are not used in ROS‐related therapeutic modalities such as PDT or SDT. Currenttly, hafnium oxide NPs (NBTXR3) that can enhance radiotherapeutic effect to soft tissue sarcoma has been approved in market, which is the first nanomedicine for ROS‐related therapy. For current ROS‐related nanosystems, there still exists several key issues in their clinical translation: i) The pathological feature varying from the living bodies may lead to the difficulties in the determination of the proper administration doses of ROS‐regulating nanomedicines. ii) Currently, a good deal of publications indicated the outstanding therapeutic effects of diverse ROS‐based nanostructures. This may bring a huge challenge in the selection of better nanosystems for follow‐up clinical evaluation. iii) In recent years, researchers tend to design nanocomposites for ROS‐based therapy. This will give rise to difficulties in investigation of the safety, mechanism and efficacy due to complex components in these nanomedicines. iv) Most of studies on ROS‐regulating nanomedicines focus on getting the nanomedicines to work. However, the study on their biological mechanisms in vivo is far from sufficient. In addition, the difference between animal models and humans makes it necessary to seek new and effective methods for safety and efficacy assessment before clinical trials. As a result, ROS‐related nanosystems for clinical translation face many challenges where more efforts are needed.

## Conflict of Interest

The authors declare no conflict of interest.

## Supporting information

Supporting Information
