# Supplementary material for: Reactive Oxygen Species‐Regulating Strategies Based on Nanomaterials for Disease Treatment
Source: Adv Sci (Weinh). 2020 Dec 20;8(3):2002797. doi: 10.1002/advs.202002797 (PMC7856897; doi:10.1002/advs.202002797)
Supplement: Supplementary file 1 — Supporting Information [file ADVS-8-2002797-s001.pdf]

WILEY-VCH

© 2020 Wiley-VCH GmbH

ADVANCED SCIENCE
Open Access

Supporting Information

for *Adv. Sci.*, DOI: 10.1002/advs.202002797

Reactive Oxygen Species-Regulating Strategies Based on Nanomaterials for Disease Treatment

Chenyang Zhang, Xin Wang, Jiangfeng Du, Zhanjun Gu, and Yuliang Zhao*

((Supporting Information can be included here using this template))

Supporting Information

Reactive Oxygen Species-Regulating Strategies Based on Nanomaterials for Disease Treatments

Chenyang Zhang, Xin Wang, Jiangfeng Du, Zhanjun Gu and Yuliang Zhao*

Dr. C. Zhang, Dr. X. Wang, Prof. Z, Gu,
CAS Key Laboratory for Biomedical Effects of Nanomaterialsand Nanosafety, Institute of High Energy Physics, Chinese Academy of Sciences,Beijing 100049, China
E-mail: zjgu@ihep.ac.cn

Dr. C. Zhang, Dr. X. Wang, Prof. Z, Gu, Prof. Y. Zhao
College of Materials Science and Optoelectronic Technology, University of Chinese Academy of Sciences, Beijing 100049, China

Prof. J. Du
First Clinical Medical College,, Shanxi Medical University, Taiyuan 030000, China

Prof. Y. Zhao
CAS Center for Excellence in Nanoscience, National Center for Nanoscience and Technology of China, Chinese Academy of Sciences, Beijing 100190, China

Prof. Y. Zhao
GBA Research Innovation Institute for Nanotechnology, Guangdong 510700, China

In order to have an objective analysis and a deep insight into the research status and current concerns about nanomaterials-mediated ROS research in biological system, we used keyword searches for the defined ROS-based nanotherapeutic modalities or nanomedicines in the field of ROS-associated antioxidant therapy, ROS-induced toxic therapy and ROS-associated nanotoxicology to search the publications in the Web of Science Core Collection database (website for providing comprehensive citation data for many different academic disciplines, Clarivate Analytics, USA). And the datas are obtained in April 19th, 2020 Beijing time.

1. ROS-based nanotherapy or nanomedicines

Drug or therap* or medic*

And reactive oxygen species OR free radical OR (superoxide or oxygen radical) OR hydroxyl radical OR hydrogen peroxide OR hypochlorous acid OR nitric oxide OR peroxynitrite

And nano* not (nano2 OR nano3 or nanolit* or nanogram* or nanomol* or nanosec*)

2. ROS-induced toxic therapy

Drug or therap* or medic*

And reactive oxygen species OR free radical OR (superoxide or oxygen radical) OR hydroxyl radical OR hydrogen peroxide OR hypochlorous acid OR nitric oxide OR peroxynitrite

And antioxida* OR protecti* OR scaveng* ROS OR eliminat* ROS OR ROS scaveng* OR ROS eliminat* OR redox homeosta* OR ((superoxide dismutase OR peroxiredoxin* OR glutathione peroxidas* OR catalase*) and deliver*)

And nano* not (nano2 OR nano3 or nanolit* or nanogram* or nanomol* or nanosec*)

3. Antioxidant therapy

Drug or therap* or medic*

And reactive oxygen species OR free radical OR (superoxide or oxygen radical) OR hydroxyl radical OR hydrogen peroxide OR hypochlorous acid OR nitric oxide OR peroxynitrite

And generat* ROS OR produc* ROS OR improv* ROS OR elevat* ROS OR ROS generat* OR ROS produc* OR ROS improv* OR ROS elevat* OR photodynamic* therap* OR radiotherap* sonodynamic therap* OR oxidative stress OR damage* OR injur* OR kill* OR death OR apoptosis OR necrosis

NOT antioxida* OR protecti*

And nano* not (nano2 OR nano3 or nanolit* or nanogram* or nanomol* or nanosec*)

4. Nanotoxicology

Drug or therap* or medic*

And reactive oxygen species OR free radical OR (superoxide or oxygen radical) OR hydroxyl radical OR hydrogen peroxide OR hypochlorous acid OR nitric oxide OR peroxynitrite

And toxicity or toxicology

NOT antioxida* OR protecti* OR photodynamic* therap* OR radiotherap* sonodynamic therap*

And nano* not (nano2 OR nano3 or nanolit* or nanogram* or nanomol* or nanosec*)
